# The Arabidopsis Mitochondrial Glutaredoxin GRXS15 Provides [2Fe-2S] Clusters for ISCA-Mediated [4Fe-4S] Cluster Maturation

**DOI:** 10.3390/ijms21239237

**Published:** 2020-12-03

**Authors:** Tamanna Azam, Jonathan Przybyla-Toscano, Florence Vignols, Jérémy Couturier, Nicolas Rouhier, Michael K. Johnson

**Affiliations:** 1 Department of Chemistry and Center for Metalloenzyme Studies, University of Georgia, Athens, GA 30602, USA; tamanna@uga.edu; 2 Université de Lorraine, INRAE, IAM, F-54000 Nancy, France; jonathan.przybyla-toscano@univ-lorraine.fr (J.P.-T.); jeremy.couturier@univ-lorraine.fr (J.C.); nicolas.rouhier@univ-lorraine.fr (N.R.); 3 BPMP, Université de Montpellier, CNRS, INRAE, SupAgro, 34060 Montpellier, France; florence.vignols@supagro.fr

**Keywords:** mitochondria, iron-sulfur protein, *Arabidopsis thaliana*, protein-protein interaction, circular dichroism, Raman spectroscopy, iron-sulfur cluster trafficking, glutaredoxin, ISCA proteins

## Abstract

Iron-sulfur (Fe-S) proteins are crucial for many cellular functions, particularly those involving electron transfer and metabolic reactions. An essential monothiol glutaredoxin GRXS15 plays a key role in the maturation of plant mitochondrial Fe-S proteins. However, its specific molecular function is not clear, and may be different from that of the better characterized yeast and human orthologs, based on known properties. Hence, we report here a detailed characterization of the interactions between *Arabidopsis thaliana* GRXS15 and ISCA proteins using both in vivo and in vitro approaches. Yeast two-hybrid and bimolecular fluorescence complementation experiments demonstrated that GRXS15 interacts with each of the three plant mitochondrial ISCA1a/1b/2 proteins. UV-visible absorption/CD and resonance Raman spectroscopy demonstrated that coexpression of ISCA1a and ISCA2 resulted in samples with one [2Fe-2S]^2+^ cluster per ISCA1a/2 heterodimer, but cluster reconstitution using as-purified [2Fe-2S]-ISCA1a/2 resulted in a [4Fe-4S]^2+^ cluster-bound ISCA1a/2 heterodimer. Cluster transfer reactions monitored by UV-visible absorption and CD spectroscopy demonstrated that [2Fe-2S]-GRXS15 mediates [2Fe-2S]^2+^ cluster assembly on mitochondrial ferredoxin and [4Fe-4S]^2+^ cluster assembly on the ISCA1a/2 heterodimer in the presence of excess glutathione. This suggests that ISCA1a/2 is an assembler of [4Fe-4S]^2+^ clusters, via two-electron reductive coupling of two [2Fe-2S]^2+^ clusters. Overall, the results provide new insights into the roles of GRXS15 and ISCA1a/2 in effecting [2Fe-2S]^2+^ to [4Fe-4S]^2+^ cluster conversions for the maturation of client [4Fe-4S] cluster-containing proteins in plants.

## 1. Introduction

Iron-sulfur (Fe-S) proteins are found in all kingdoms of life and are involved in fundamental biological processes, ranging from electron transfer and catalyzing metabolic reactions to small molecule sensing, DNA repair and regulating gene expression [1,2,3]. Synthetic and biological Fe-S clusters can generally be assembled via spontaneous self-assembly using excess iron and sulfide ions under anaerobic conditions. However, having these moieties in excess would be toxic to cells. Therefore, cells use complex maturation systems to ensure the correct assembly, trafficking and insertion of Fe-S clusters [1,4,5].

The mitochondrial iron-sulfur cluster (ISC) assembly machinery is conserved in most eukaryotes and shares components with the bacterial ISC machinery due to the evolutionary relationship between bacteria and mitochondria [6,7]. Moreover, in addition to the biosynthesis of Fe-S clusters in mitochondrial proteins, the ISC machinery is also required for the maturation of cytosolic and nuclear Fe-S proteins [8]. Based on studies performed in yeast and humans, mitochondrial Fe-S cluster biogenesis can be divided into four steps: (i) cysteine desulfurase-mediated assembly of a [2Fe-2S]^2+^ cluster on an ISU/ISCU scaffold protein, (ii) molecular chaperone-assisted [2Fe-2S]^2+^ cluster transfer from ISU to acceptor proteins, (iii) synthesis of [4Fe-4S]^2+^ clusters from [2Fe-2S]^2+^ clusters, and (iv) intact [4Fe-4S]^2+^ cluster transfer to client proteins.

The early steps of mitochondrial ISC machinery involve biosynthesis (step 1) and trafficking (step 2) of [2Fe-2S]^2+^ clusters [5]. In yeast, the proteins involved with the biosynthetic complex include Nfs1, Isd11, acyl carrier protein 1 (Acp1), scaffold proteins Isu1/2, ferredoxin (Yah1) and frataxin (Yfh1). Yeast deletion mutant studies and structural analyses of protein complexes have notably been instrumental in characterizing the roles of these proteins [8]. The scaffold role is performed by Isu1 in yeast (the bacterial IscU ortholog) [9]. In arabidopsis, there are three mitochondrial-located ISU proteins, namely ISU1, ISU2, and ISU3, but ISU1 is likely to be the primary scaffold protein [10,11,12]. A pyridoxal 5′-phosphate (PLP)-dependent cysteine desulfurase, Nfs1/NFS1 in yeast/human, converts cysteine to alanine and incorporates the released S as a cysteine persulfide on a flexible loop, which can be transferred to form a cysteine persulfide on Isu1 via a disulfide exchange mechanism [13]. In humans, maturation of [2Fe-2S]^2+^ clusters on ISCU2 occurs in a complex that involves NFS1, ISD11, ACP1 and ISCU2 as core proteins and ferredoxin 2 (FDX2) and frataxin (FXN) as transient participants [5,14,15,16]. Counterparts are present in plant mitochondria, although the components of the core assembly complex have yet to be confirmed. In yeast, the chaperone Ssq1 and J-type cochaperone Jac1 work together for efficient transfer of [2Fe-2S]^2+^ clusters from Isu1 to monothiol glutaredoxin 5 (Grx5), or other apo-acceptor proteins, in an ATP-dependent process [5,17,18,19]. *At HSCA1* and *At HSCB* complement the yeast knockout strains for the respective *Ssq1* and *Jac1* orthologs [20].

While the early ISC machinery appears to provide the [2Fe-2S]^2+^ cluster building blocks for [4Fe-4S]^2+^ cluster biogenesis, the molecular mechanisms of [4Fe-4S]^2+^ cluster assembly, trafficking, and insertion into target proteins are less well understood. The existing body of research has led to two hypotheses regarding [4Fe-4S]^2+^ cluster assembly. The evidence for facile reductive coupling of two [2Fe-2S]^2+^ clusters to form a [4Fe-4S]^2+^ cluster at the subunit interface of bacterial IscU dimers, that could be used to activate apo-aconitase [21], raised the possibility that [4Fe-4S]^2+^ clusters can be assembled on U-type scaffold proteins and transferred directly to a [4Fe-4S]^2+^ cluster carrier or acceptor proteins. This hypothesis has recently been supported by nuclear magnetic resonance (NMR), small-angle X-ray scattering (SAXS) and isothermal titration calorimetry (ITC) studies which show interaction between human ISCU and NFU1 and [4Fe-4S]^2+^ cluster transfer from ISCU to NFU1 [22]. The second hypothesis is that [4Fe-4S]^2+^ clusters are formed on A-type carrier (ATC) proteins via [2Fe-2S]^2+^ cluster transfer from a monothiol GRX. This was originally proposed based on the observation of facile and reversible [2Fe-2S]^2+^ to [4Fe-4S]^2+^ cluster interconversions on ^Nif^IscA [23]. Additional support for this proposal was provided by the observation that two human GLRX5 homodimers donate [2Fe-2S]^2+^ clusters to form a [4Fe-4S]^2+^ cluster on a ISCA1-ISCA2 heterodimeric complex [24,25].

By analogy with the bacterial and yeast ISC systems [18,19], a monothiol GRX is likely to be the dedicated acceptor protein for [2Fe-2S]^2+^ clusters assembled on ISU1 in plants. GRXS15 is the sole monothiol GRX in arabidopsis mitochondria, and it has a 33% amino acid similarity with yeast and human mitochondrial monothiol GRXs (Figure S1). The involvement of Grx5 and its orthologs in mitochondrial Fe-S cluster assembly has been established in yeast and vertebrates [9,26], but has only recently been investigated in plants [27,28]. The arabidopsis *grxs15* null mutants are not viable indicating an essential role for plant viability, and GRXS15 was found to assemble an Fe-S cluster in vitro in the presence of glutathione [27]. Targeted mutagenesis studies of *At GRXS15* resulted in variants with diminished glutathione and Fe-S cluster content. Using these variants for complementing arabidopsis *grxs15* lines led to a dwarf phenotype and a 65% decrease in aconitase activity [27]. The study of RNAi lines for *At GRXS15* also pointed to a defect in lipoic-acid synthesis [28]. These findings indicated that *At* GRXS15 is an essential protein that is involved in the maturation of at least some mitochondrial Fe-S proteins in plants.

Gene knockdown studies indicate that a complex composed of Isa1-Isa2-Iba57 and ISCA1-ISCA2-IBA57 in yeast and human cell lines, respectively, is required for the maturation of [4Fe-4S] proteins but not [2Fe-2S] proteins [29,30,31]. This complex does not react with early ISC machinery but rather depends on the delivery of [2Fe-2S]^2+^ clusters from Grx5/GLRX5. However, more recent studies with mammalian systems suggest greater functional complexity for ISCA complexes. For example, in vitro studies showed that only the heterodimeric ISCA1-ISCA2 complex, and not individual ISCA1 or ISCA2 homodimers, formed a [4Fe-4S]^2+^ cluster by [2Fe-2S]^2+^ cluster transfer from GLRX5 [24,32]. This result casts doubt on the need for *Hs* IBA57 for [4Fe-4S]^2+^ cluster assembly on the *Hs* ISCA1/2 heterodimer [24]. Moreover, *Hs* ISCA2, but not *Hs* ISCA1, was found to react with *Hs* IBA57 forming a [2Fe-2S]^2+^ bound heterocomplex [33,34]. Previous in vivo studies indicate that both *Sc* Isa1 and *Sc* Isa2 interact with *Sc* Iba57 [31]. In addition, mouse skeletal muscle and neuronal gene knockdown experiments suggest that ISCA1, but not ISCA2, is required for [4Fe-4S] cluster incorporation on acceptor proteins [35]. Taken together, these results demonstrate that much has still to be learned about the roles of mitochondrial ATC and IBA57 proteins.

Arabidopsis has two IBA57 and four ATC proteins: IBA57.2 and SUFA1 are localized in plastids while IBA57.1 and the other three ATC (i.e., ISCA1a, ISCA1b and ISCA2) are (or are predicted to be) present in mitochondria [36,37,38]. *At* ISCA1a and *At* ISCA1b are orthologs of yeast Isa1 and human ISCA1 [39], and *At* ISCA2 is an ortholog of yeast Isa2 and human ISCA2 [40]. Hence the presence of two ISCA1 orthologs in plants adds additional complexity compared to mammals. However, all three conserved cysteines responsible for ligation of the Fe-S cluster are present in each of the three arabidopsis mitochondrial ATC proteins (Figure S2).

Although a model of the mitochondrial ISC machinery has been proposed [8], there are still many uncertainties concerning the molecular mechanisms supporting the late steps. Moreover, mechanistic information concerning plant ISC machinery is almost nonexistent. This is due notably to the complexity arising from the presence of multiple copies of several key proteins, e.g., three scaffold proteins (ISU1/2/3), two ferredoxins (mFDX1/2), two sets of chaperones (HSCA1/2) and nucleotide exchange factors (MGE1a/b), two NFU-type cluster transfer proteins (NFU4/5) and three ISCA-type cluster transfer proteins (ISCA1a/1b/2) [6]. Consequently, the majority of the proteins involved in the late steps of the plant mitochondrial ISC machinery, i.e., acting after GRXS15, have neither been functionally analyzed using plant mutants nor purified and characterized in vitro. Recent complementation studies performed with *At* NFU4/5 and ISCA1/2 and the corresponding yeast deletion mutants reported that *At* NFU4/5 perform similar functions to yeast Nfu1 and that the heterodimeric complex *At* ISCA1a/2 can functionally substitute for the yeast Isa1/2 proteins, while *At* ISCA1a or *At* ISCA2 homodimers cannot [39,41,42]. These observations suggest that the heterodimeric ISCA1a/2 complex is the functional unit in the mitochondrial ISC machinery, in agreement with the in vitro studies performed with human proteins [24]. Considering the conservation of these steps, it is puzzling that GRXS15 from poplar or arabidopsis fail to complement most defects of the corresponding yeast *grx5* mutant [27,43].

The objectives of this work were to clarify the function of late-acting ISC proteins in plants by investigating the cellular interactions between the monothiol GRXS15 and ISCA proteins and the properties and cluster transfer reactivity of the corresponding recombinant proteins. The results show that (i) GRXS15 interacts with all three ISCA proteins, (ii) recombinant ISCA1a/2 heterodimer contains one [2Fe-2S]^2+^ cluster as-purified, which is converted to a [4Fe-4S]^2+^ cluster by anaerobic cluster reconstitution and (iii) [2Fe-2S]^2+^ GRXS15 can assemble a [4Fe-4S]^2+^ cluster on ISCA1a/2 heterodimer in the absence of IBA57.

## 2. Results

### 2.1. Interaction of GRXS15 with ISCA Proteins

**Figure 1:**
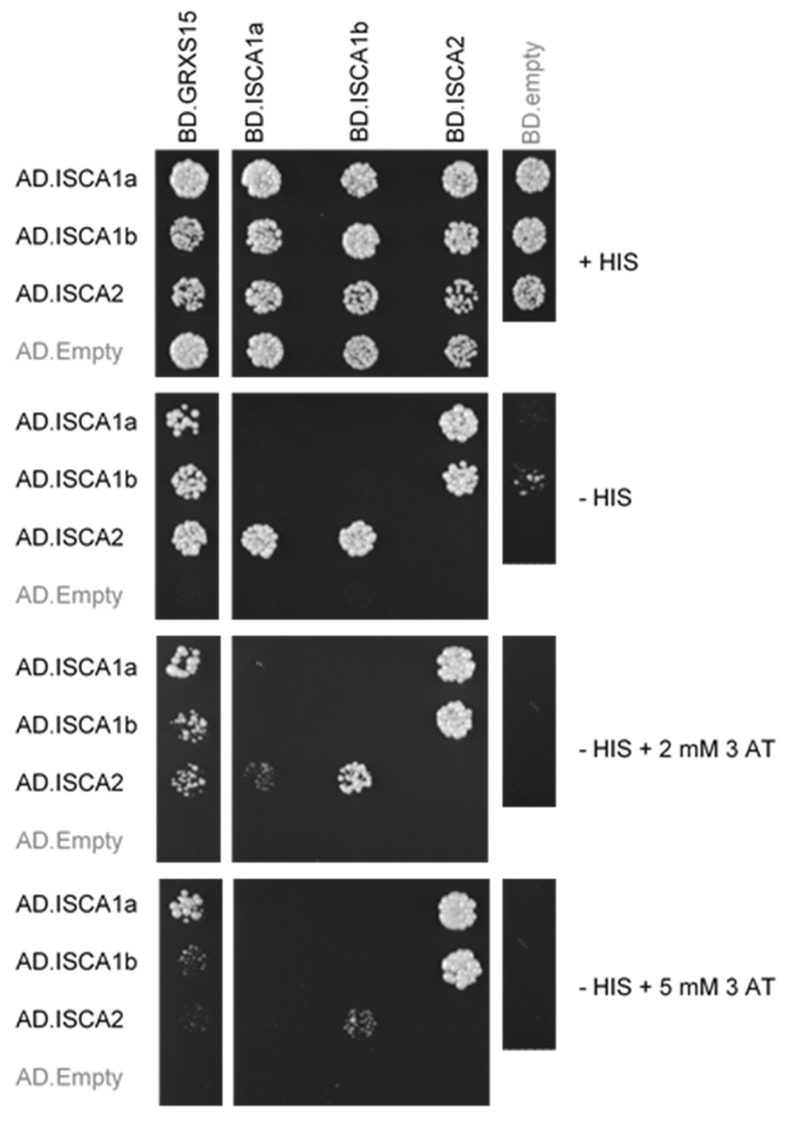
**Interactions among arabidopsis GRXS15 and ISCA proteins detected by binary yeast two-hybrid assays.** The cotransformed yeast cells were plated at an OD_600_ of 0.05 on a control plate containing histidine (+HIS) and on test plates without histidine (-HIS) and eventually containing 2 or 5 mM 3-amino-1,2,4-triazole (3AT). The constructs allow expressing fusions between the Gal4 activation domain (AD) or Gal4 DNA binding domain (BD) and the mature forms of each protein at the C-terminus. Yeast growth revealing protein-protein interaction was recorded after five days at 30 °C. Yeast cells were also cotransformed with plasmid pairwises involving an empty pGADT7 or pGBKT7 (AD or BD-empty) to check that none of the proteins expressed alone in yeast cells can transactivate the HIS reporter gene. Only the AD-ISCA1a construct generated a weak transactivation (i.e., visible only in absence of 3-AT). The images shown are representative of three independent transformation experiments.

The current model of the ISC machinery in non-photosynthetic organisms indicates that mitochondrial monothiol Grx5 is the immediate cluster donor to Isa/ISCA proteins [8]. In order to test whether *At* GRXS15 does interact with all three mitochondrial *At* ISCA isoforms (i.e., ISCA1a, ISCA1b, ISCA2), we used a Gal4-based binary yeast two-hybrid (Y2H) experiment. To prevent autoactivation and to test the strength of the interactions, 3-amino-1,2,4-triazole (3-AT) was added at a concentration of 2 mM or 5 mM. Upon coexpression in yeast cells, GRXS15 was found to interact with all ISCA proteins when fused to the DNA binding domain of Gal4, whereas no interaction was detected when it was fused to the Gal4-activation domain (Figure 1). Concerning ISCA proteins, ISCA1a and ISCA1b interacted with ISCA2 regardless of the type of fusion tested on 3-AT concentrations up to 5 mM (Figure 1). No evidence for the formation of ISCA1a, ISCA1b and ISCA2 homodimers or ISCA1a/ISCA1b heterodimers was obtained using this method. These results indicated that *A. thaliana* ISCA1/2 heterodimers are formed in a cellular yeast system, supporting previous in vitro observations [24,40].

**Figure 2:**
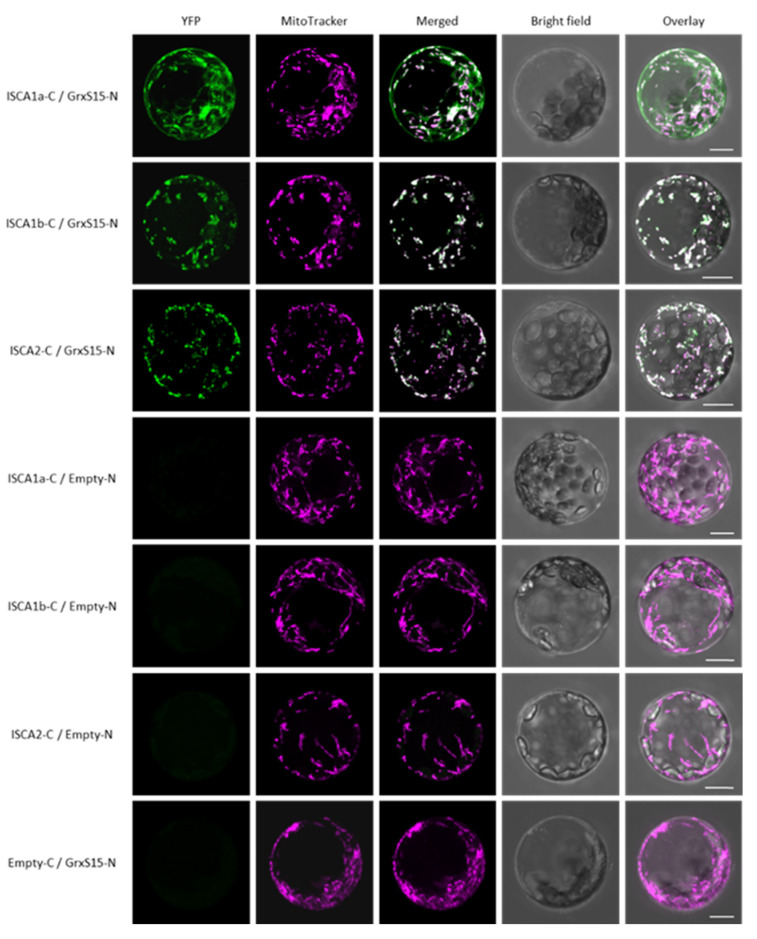
**BiFC assays between arabidopsis GRXS15 and ISCA proteins in arabidopsis leaf protoplasts.** Arabidopsis protoplasts obtained from four week-old plantlets were transfected with combinations of two vectors expressing GRXS15 fused to the N-terminal region of YFP (GRXS15-N in panels) and ISCAs cloned upstream of the C-terminal region of YFP (ISCA-C in panels). The YFP fluorescence was recorded 24 h post-transfection by confocal microscopy. All confocal images shown here were captured using a maximum Z-stack intensity projection. Images showing confocal plans without Z-stack intensity projection are shown in Figure S3. Negative controls verifying that none of the proteins expressed alone can restore YFP fluorescence are shown. BiFC results obtained using opposite protein fusion conformations (GRXS15-C coexpressed with ISCA-N) showed less clear-cut results as a strong fluorescence in the cytosol suggested the formation of aggregates was obtained for some combinations. Bars = 10 μm.

To confirm these interactions in plant cells, we additionally performed bimolecular fluorescence complementation (BiFC) assays in arabidopsis protoplasts using GRXS15 fused upstream of the N-terminal domain of the yellow fluorescent protein (YFP) protein and ISCA proteins fused upstream of the C-terminal region of YFP (GRXS15-N and ISCA-C in Figure 2, respectively). Positive BiFC signals confirmed that GRXS15 is in the close environment of the three ISCAs within plant cells, and that these GRXS15/ISCA interactions occur in mitochondria (Figure 2 and Figure S3). Concerning the GRXS15/ISCA1a couple, an additional faint YFP signal was also observed in the cytosol of all cells analyzed, very likely arising from the overexpression levels which affect a correct localization (Figure 2 and Figure S3, first lane). Regarding ISCA1/2 heterodimers, their validation by BiFC could not be assessed because of aspecific BiFC patterns (i.e., strong fluorescence in the cytosol likely due to protein mistargeting or formation of aggregates).

### 2.2. Nature and Properties of Fe-S Clusters Assembled on Arabidopsis thaliana GRXS15

**Figure 3:**
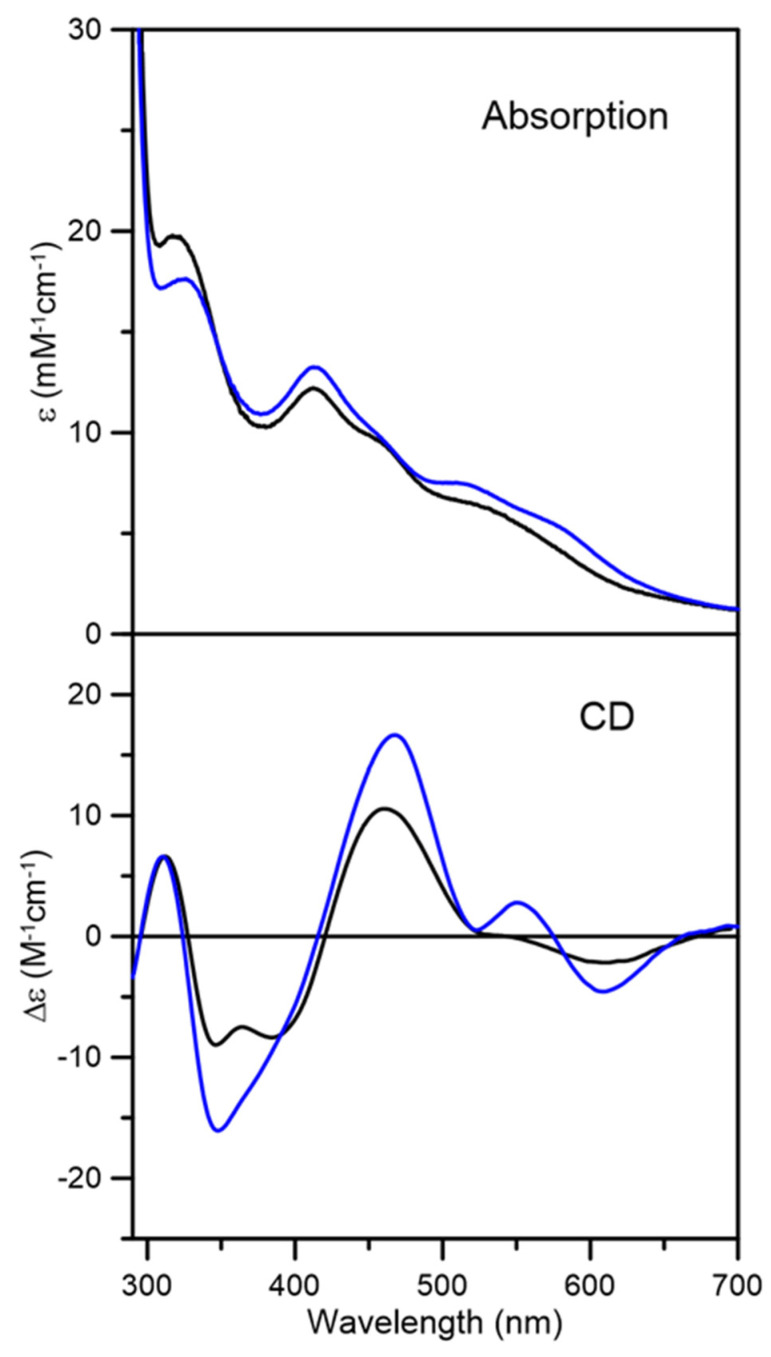
**UV-visible absorption and circular dichroism spectra of reconstituted *At* GRXS15.** Fraction 1 (black line) and fraction 2 (blue line) were obtained after separation using a Mono-Q column. Spectra were recorded under anaerobic conditions in sealed 0.1 cm cuvettes in 100 mM Tris-HCl buffer with 5 mM GSH at pH 7.5. The ε and Δε values are based on concentration of *At* GRXS15 dimer.

Aerobic purification of *At* GRXS15 in the presence of GSH resulted in a brown fraction with weak visible absorption characteristic of trace amounts of a [2Fe-2S]^2+^ cluster (data not shown). Consequently, samples of cluster-bound *At* GRXS15 for spectroscopic characterization and cluster transfer studies were prepared by reconstituting apo-GRXS15 under anaerobic conditions. No Fe-S cluster was assembled in samples reconstituted in the absence of GSH, either after pretreatment with DTT or in the presence of 5 mM DTT. This is in contrast to *Sc* Grx5, which reconstituted [4Fe-4S]^2+^ clusters in the presence of 5 mM DTT [44]. However, in vitro IscS-mediated reconstitution of apo-GRXS15 in the presence of 5 mM GSH, resulted in samples containing a mixture of [2Fe-2S]^2+^ and linear [3Fe-4S]^1+^ clusters, based on comparison of UV-visible absorption and CD spectra with published data for other cluster-bound monothiol GRXs [44]. Purification of the reconstitution mixture using a Mono-Q column removed excess reagents and separated the homogeneous [2Fe-2S]^2+^ cluster-bound form of GRXS15 from the linear [3Fe-4S]^1+^ cluster-bound form. The UV-visible absorption and CD spectra of fraction one were very similar to those of the [2Fe-2S]^2+^-bound *At* GRXS16 and *Sc* Grx3 [43,45,46], indicating homogenous [2Fe-2S]^2+^ cluster-bound GRXS15 (Figure 3). Moreover, Fe and protein concentrations indicated 2.1 ± 0.2 Fe/dimer, in accord with one [2Fe-2S]^2+^ cluster per dimer. The UV-visible absorption and CD spectra of the other fraction, notably the general increase in absorption intensity and appearance of pronounced bands at 518 nm and 570 nm, coupled with the appearance of a positive CD band at 550 nm, are indicative of the presence of linear [3Fe-4S]^1+^ clusters [44,45,46,47], see Figure 3. The UV-visible absorption and CD spectra of this fraction are best interpreted as containing a mixture of [2Fe-2S]^2+^ and linear [3Fe-4S]^1+^ clusters. In this work, only the fraction containing homogenous [2Fe-2S]^2+^ cluster was used for further studies.

### 2.3. In Vitro Cluster Transfer from [2Fe-2S]^2+^-GRXS15 to Mitochondrial Apo-FDX1

**Figure 4:**
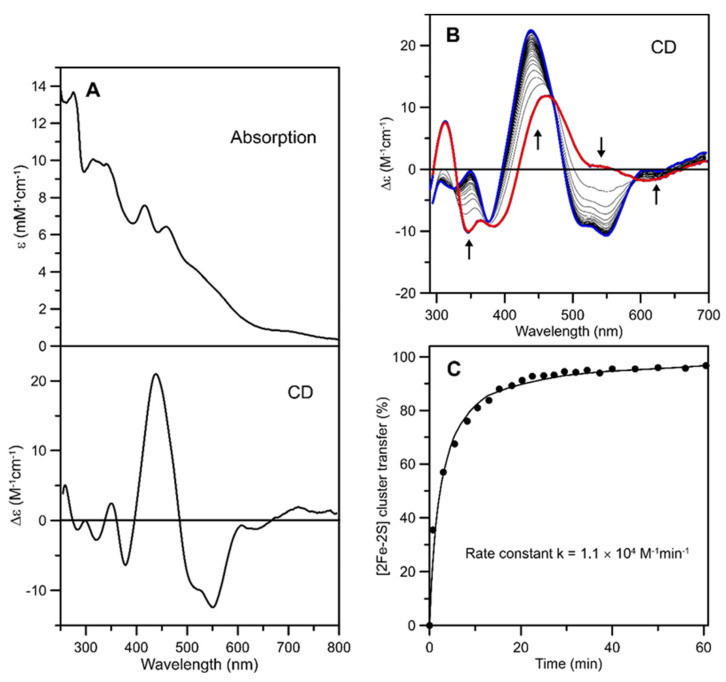
**Cluster transfer from *At* [2Fe-2S]^2+^-GRXS15 to *At* apo-mFDX1 monitored by CD spectroscopy as a function of time.** (**A**) Room temperature UV-visible absorption and CD spectra of [2Fe-2S]^2+^ cluster-bound as-isolated *At* mFDX1. (**B**) CD spectra of the cluster transfer reaction mixture that was initially 40 µM in GRXS15 [2Fe-2S]^2+^ clusters and 40 µM in apo-mFDX1. The thick red line corresponds to [2Fe-2S]^2+^-GRXS15 recorded before addition of apo-mFDX1. The thin grey lines correspond to CD spectra recorded at 1, 3, 5, 8, 10, 13, 15, 17, 20, 22, 25, 27, 30, 32, 34, 37, 40, 45, 50, 56, 60, 69, 75, 86, 95, 101, 105, 110 and 120 min after the addition of apo-mFDX1. The thick blue line corresponds to complete [2Fe-2S]^2+^ cluster transfer to mFDX1. The arrows indicate the direction of intensity change with increasing time at selected wavelengths and Δε values were calculated based on the initial concentration of [2Fe-2S]^2+^ clusters. The cluster transfer reaction was carried out under anaerobic conditions at room temperature in 100 mM Tris-HCl buffer at pH 7.8. (**C**) Kinetic simulation of cluster transfer from [2Fe-2S]^2+^-GRXS15 to apo-mFDX1 based on second-order kinetics and the initial concentrations of [2Fe-2S]^2+^ clusters on [2Fe-2S]^2+^-GRXS15 and of apo-mFDX1. Percent cluster transfer was assessed by CD intensity at 550 nm (black circles) and simulated with a second-order rate constant of 1.1 × 10^4^ M^−1^min^−1^.

Previous studies have reported that the chloroplastic monothiol GRXS14 and GRXS16 can donate [2Fe-2S]^2+^ clusters to chloroplastic apo-ferredoxin [43,48]. Aerobically-purified mitochondrial FDX1 (mFDX1) exhibited UV-visible absorption and CD spectra characteristics of a [2Fe-2S]^2+^ protein. The spectra shown in Figure 4A are very similar to those of purified human mitochondrial ferredoxins, *Hs* FDX1 and *Hs* FDX2 [49], and Fe and protein concentrations indicated 0.89 ± 0.20 [2Fe-2S] clusters per monomer. *At* mFDX1 was used to assess the ability of *At* GRXS15 to function as a cluster transfer protein for maturation of [2Fe-2S] cluster-containing client proteins, since the holo-forms of both proteins have major differences in their CD spectra, see Figure 4B. *At* apo-mFDX1 was prepared by acid precipitation with 10% trichloroacetic acid followed by resuspension in 100 mM Tris-HCl buffer at pH 7.8. Anaerobic cluster transfer from [2Fe-2S]^2+^-GRXS15 to DTT-pretreated apo-mFDX1 with a 1:1 donor:acceptor ratio was 90% complete after 22 min and showed one set of isodichroic points, indicating intact cluster transfer (Figure 4B). Percent cluster transfer was assessed by CD intensity at 550 nm (black circles) and simulated with a second-order rate constant of 1.1 × 10^4^ M^−1^min^−1^ based on initial 40 μM concentrations for donor and acceptor (Figure 4C). Control studies showed no reaction for the reverse cluster transfer (see Figure S4), indicating a unidirectional reaction. Moreover, the observation of no degradation of the [2Fe-2S]^2+^ cluster on GRXS15 in the absence of apo-mFDX1 over the time course of the reaction is in accord with intact cluster transfer. Clearly, GRXS15 is an effective [2Fe-2S]^2+^ cluster donor for rapid and quantitative maturation of mFDX1.

### 2.4. Purification and Spectroscopic Characterization of Fe-S Clusters Assembled on At ISCA1a/2

**Figure 5:**
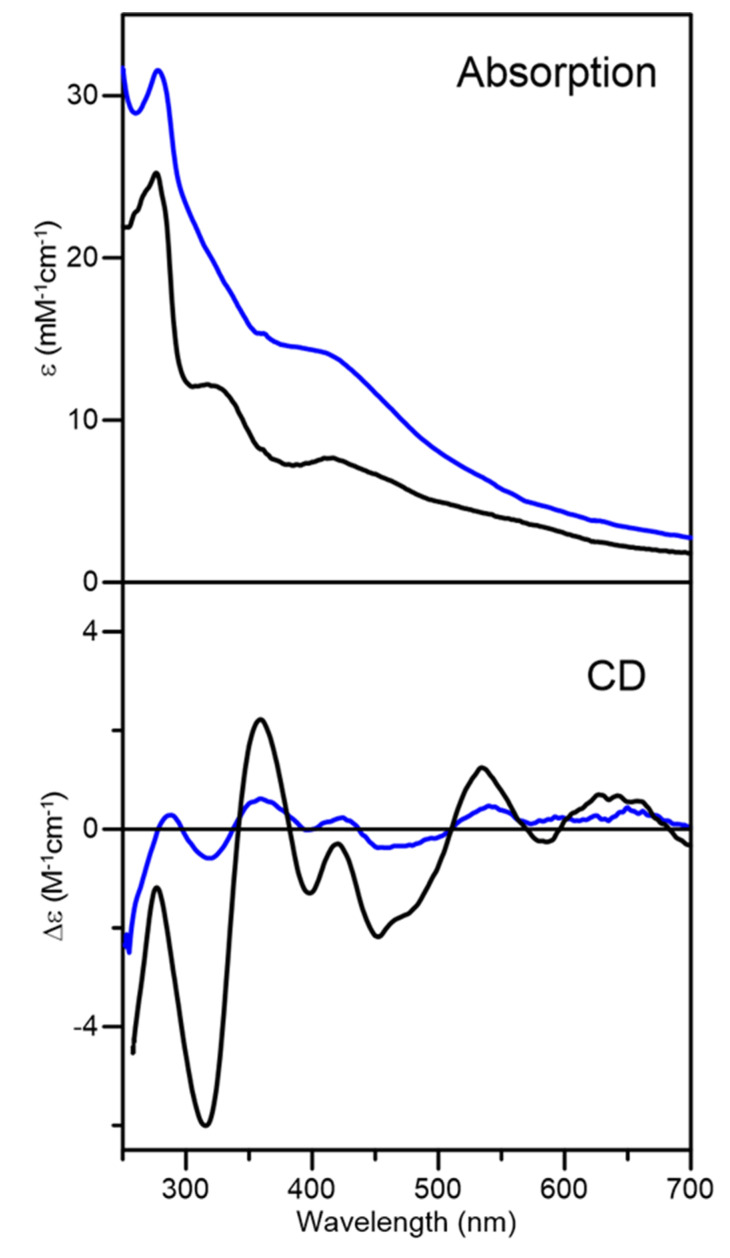
**Room temperature UV-visible absorption and CD spectra of *At* ISCA1a/2 heterodimer.** The [2Fe-2S]^2+^ cluster-bound as-isolated *At* ISCA1a/2 (black lines) and [4Fe-4S]^2+^ cluster-bound reconstituted *At* ISCA1a/2 (blue lines) are shown. All ε and Δε values are based on ISCA1a/2 heterodimer concentration.

Based on the fact that *At* ISCA1b was produced as an insoluble protein in *Escherichia coli*, we have only coexpressed *At* ISCA1a/2. Purification under strictly anaerobic conditions resulted in a reddish-brown [2Fe-2S]^2+^ cluster-bound heterodimeric form of ISCA1a/2 (Figure S5). The UV-visible absorption spectrum of anaerobically purified ISCA1a/2 has resolved bands centered at 320 nm and 415 nm, and shoulders at ~455 nm and ~530 nm, and the CD spectrum has positive bands centered at 359 nm and 543 nm and negative bands centered at 304 nm, 453 nm, and 606 nm (Figure 5, black lines). Both spectra are characteristic of [2Fe-2S]^2+^ clusters found in ATC proteins [23,46]. Protein and iron analysis indicated 0.83 ± 0.10 [2Fe-2S]^2+^ clusters per heterodimeric ISCA1a/2. Since [2Fe-2S]^2+^ clusters typically have ε_425_ values between 7 and 11 mM^−1^cm^−1^, and the UV-visible absorption spectrum of ISCA1a/2 indicated ε_425_ = 7.2 ± 0.2 mM^−1^cm^−1^, this result also indicated that as-purified coexpressed ISCA1a/2 contains one [2Fe-2S]^2+^ cluster per heterodimer.

**Figure 6:**
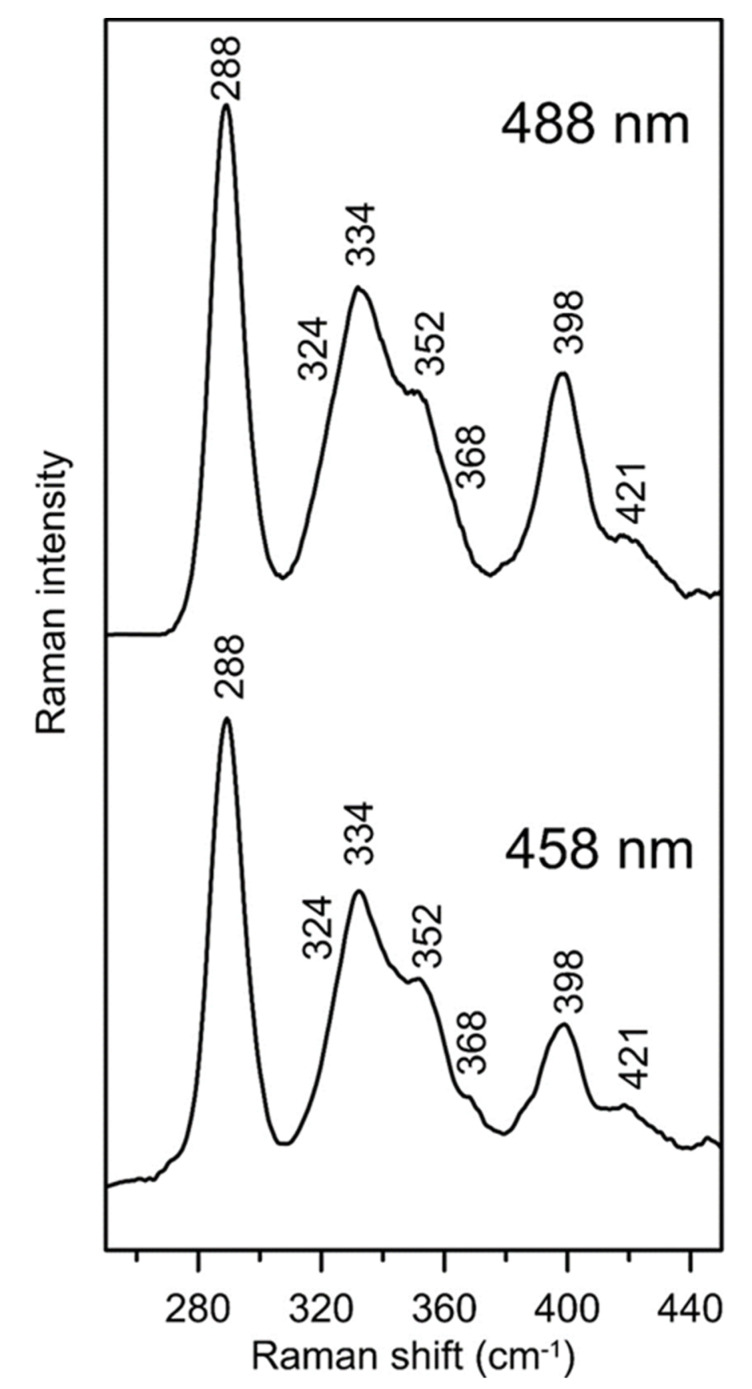
**Resonance Raman spectra of [2Fe-2S]^2+^ cluster-bound as-isolated *At* ISCA1a/2 using 458-nm and 488-nm laser excitation**. The sample (~2 mM [2Fe-2S]^2+^ clusters) in 100 mM Tris-HCl buffer at pH 7.8 was in the form of a frozen droplet at 17 K. The spectrum is the sum of 100 individual scans with each scan involving photon counting for 1 s at 0.5 cm^−1^ increments with 7 cm^−1^ spectral resolution. Bands due to lattice modes of the frozen buffer solution were subtracted from both spectra.

The vibrational properties of the [2Fe-2S]^2+^ center in ISCA1a/2 were characterized by resonance Raman spectroscopy, which provides information on cluster type and ligand environment. Resonance Raman spectra of as-purified [2Fe-2S]^2+^ cluster-bound ISCA1a/2 in the Fe-S stretching region (240–450 cm^−1^), obtained using 458 and 488 nm laser excitation, showed an intense band at 288 cm^−1^ and additional major bands at 334 cm^−1^, 352 cm^−1^, 398 cm^−1^ and 421 cm^−1^ (Figure 6). The Fe-S stretching frequencies for the [2Fe-2S]^2+^ cluster in ISCA1a/2 are very similar to those of the all-cysteine ligated [2Fe-2S]^2+^ centers in chloroplastic ferredoxins and the *Azotobacter vinelandii* (*Av*) ^Nif^IscA homodimer [23,50,51], indicating similar cluster ligation in ISCA1a/2. The frequencies are readily assigned to the stretching modes of the Fe_2_S^b^_2_S^t^_4_ center (S^b^ = bridging S and S^t^ = terminal or cysteinyl S), under idealized *D_2h_* symmetry, based on published normal mode calculations and ^34^S^b^/^32^S^b^ isotope shifts for synthetic and biological [2Fe-2S]^2+^ clusters [23,50], see Table 1.

A [4Fe-4S]^2+^ cluster-containing form of homodimeric *Av* ^Nif^IscA can be generated by incubating the subunit-bridging [2Fe-2S]^2+^ cluster-containing form with DTT for 15 min under anaerobic conditions [23]. A dissociative mechanism involving two-electron reductive coupling of two [2Fe-2S]^2+^ clusters at the subunit interface of ^Nif^IscA monomers was proposed for the formation of a subunit bridging [4Fe-4S]^2+^ cluster. Subsequent exposure to O_2_ resulted in oxidative cleavage of the [4Fe-4S]^2+^ cluster-bound form of homodimeric ^Nif^IscA to form the original [2Fe-2S]^2+^ cluster-bound form. This suggested that rapid and reversible interconversion between two [2Fe-2S]^2+^ clusters and one [4Fe-4S]^2+^ cluster can occur on ^Nif^IscA in a dithiol reducing medium, with the reaction direction determined by the level of oxidative stress [23]. However, unlike the [2Fe-2S]^2+^ cluster-bound ^Nif^IscA homodimer, [2Fe-2S]^2+^ cluster-bound ISCA1a/2 heterodimer did not undergo conversion to a [4Fe-4S]^2+^ cluster-bound form upon anaerobic incubation with 5 mM DTT for 30 min as judged by unchanged absorption and CD spectra (data not shown). This observation is similar to the one made for human ISCA1 or ISCA2 homodimers [25,32].

**Figure 7:**
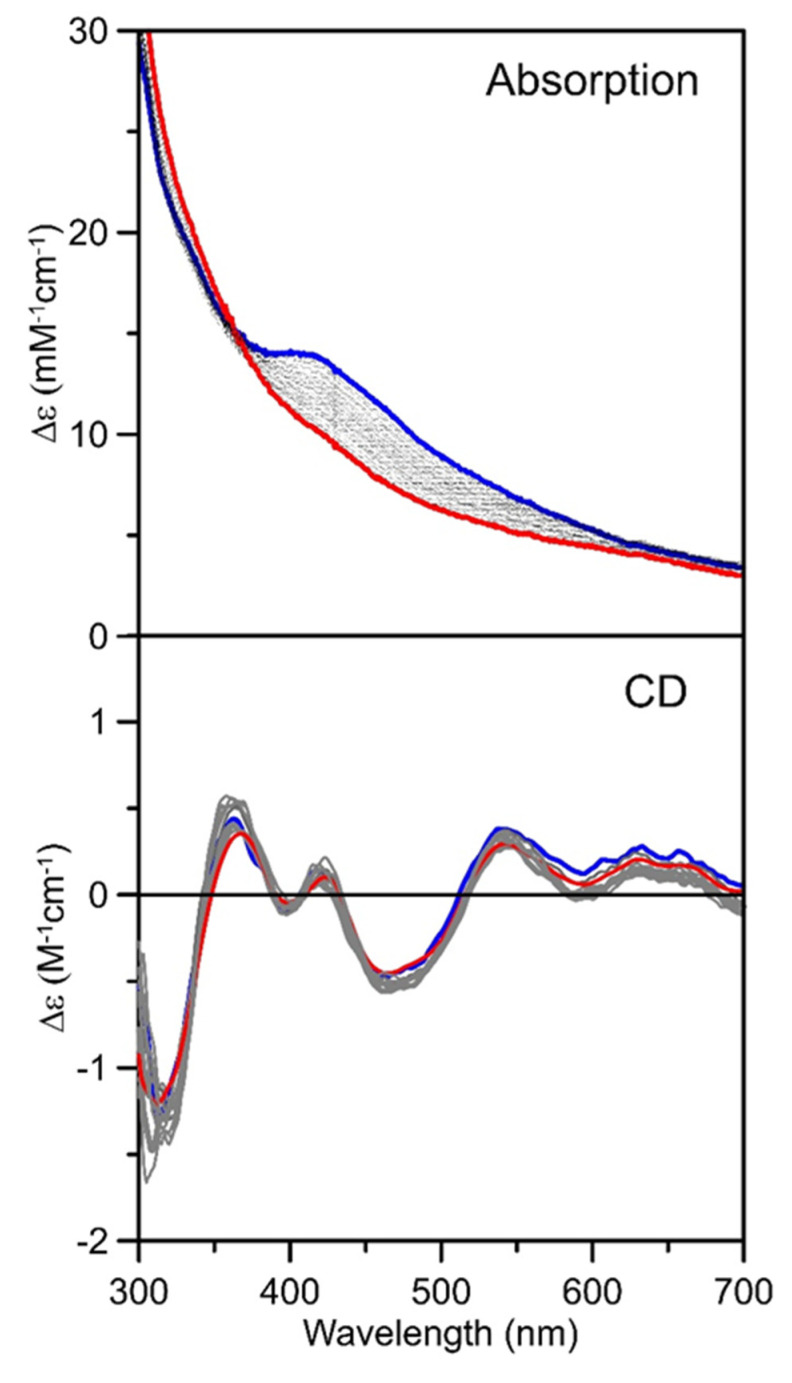
**Oxygen degrades [4Fe-4S]^2+^ clusters, but not [2Fe-2S]^2+^ clusters, in reconstituted *At* ISCA1a/2.** Anaerobically-reconstituted [4Fe-4S]^2+^ cluster-bound (thick blue lines) was monitored in 1 cm cuvettes by UV-visible absorption and CD for 180 min after exposure to air (thin gray lines). The final spectra recorded after 200 min is shown as thick red lines. The CD spectra only monitor oxygen-induced changes in the minority (20%) [2Fe-2S]^2+^ cluster-bound form of ISCA1a/2, since the majority (80%) [4Fe-4S]^2+^ cluster-bound form does not exhibit a significant CD spectrum. All ε and Δε values are based on ISCA1a/2 heterodimer concentration.

In contrast, anaerobic IscS-mediated reconstitution of the as-purified [2Fe-2S]^2+^ cluster-bound ISCA1a/2 heterodimer, in the presence of excess ferrous ammonium sulfate (FAS) and L-cysteine, and 2 mM DTT, resulted in a predominantly [4Fe-4S]^2+^ cluster-bound ISCA1a/2 heterodimer (see Figure 5, blue lines). The UV-visible absorption spectrum of reconstituted ISCA1a/2 showed broad shoulders centered near 320 and 400 nm (Figure 5), which are indicative of a [4Fe-4S]^2+^ cluster. Protein and iron analyses revealed 3.6 ± 0.4 Fe per heterodimer and the extinction coefficients based on the protein heterodimer, ε_280_ = 32 mM^−1^cm^−1^ and ε_420_ = 15 mM^−1^cm^−1^, indicate approximately one [4Fe-4S]^2+^ cluster per heterodimer. As for the [4Fe-4S]^2+^ cluster-bound ^Nif^IscA homodimer [23], [4Fe-4S]^2+^ cluster-bound ISCA1a/2 appears to have negligible visible CD intensity and the observed CD spectrum clearly results from residual [2Fe-2S]^2+^ cluster-bound ISCA1a/2, which accounts for approximately 20% of cluster-bound ISCA1a/2 based on the Δε values. This was confirmed, along with direct evidence for the O_2_-sensitivity of [4Fe-4S]^2+^ centers, but not [2Fe-2S]^2+^ centers, in ISCA1a/2, by UV-visible absorption and CD studies of the effect of air-exposure on reconstituted ISCA1a/2, see Figure 7. The gradual loss of the shoulder at 400 nm in the absorption spectrum after 200 min of air exposure indicates oxidative degradation of the [4Fe-4S]^2+^ cluster. Moreover, the lack of change in the CD spectrum demonstrates that the residual [2Fe-2S]^2+^ is resistant to oxidative degradation.

**Figure 8:**
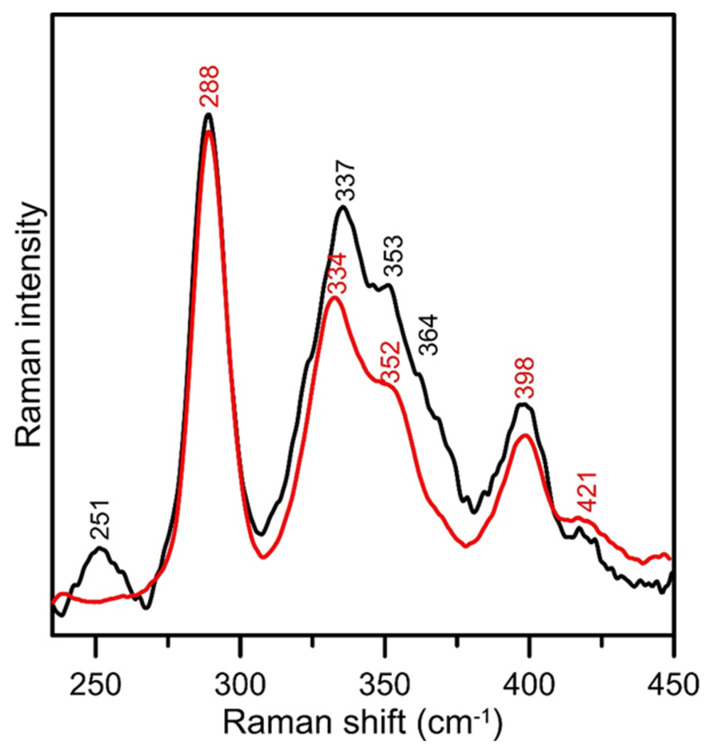
**Comparison of the resonance Raman spectra of [2Fe-2S]^2+^ cluster-bound as-isolated *At* ISCA1a/2 (red line) and [4Fe-4S]^2+^ cluster-bound reconstituted *At* ISCA1a/2 (black line) using 458-nm laser excitation.** The samples (~2 mM [2Fe-2S]^2+^ or [4Fe-4S]^2+^ clusters) in 100 mM Tris-HCl buffer at pH 7.8 were in the form of frozen droplets at 17 K. The spectra are the sum of 100 individual scans with each scan involving photon counting for 1 s at 0.5 cm^−1^ increments with 7 cm^−1^ spectral resolution. Bands due to lattice modes of the frozen buffer solution have been subtracted from both spectra.

Confirmation of the formation of [4Fe-4S]^2+^ clusters in reconstituted ISCA1a/2 was provided by resonance Raman. As noted previously, resonance enhancements of Fe-S stretching modes associated with [2Fe-2S]^2+^ centers are approximately 10 times greater than for [4Fe-4S]^2+^ centers using 458 nm excitation, due to the increased ferric character in [2Fe-2S]^2+^ clusters [52]. Consequently, the 20% occupancy of residual [2Fe-2S]^2+^ clusters dominates the resonance Raman spectrum of reconstituted ISAC1a/2. However, the dominant bands expected for a [4Fe-4S]^2+^ center are apparent by comparing the spectra of as-purified and reconstituted ISCA1a/2 after normalizing the photon counts at 288 cm^−1^, which corresponds to the most intense band of the [2Fe-2S]^2+^ center, see Figure 8. Reconstituted ISCA1a/2 has additional bands at 251 cm^−1^ (asymmetric *T_2_* ν(Fe−S*^b^*)), 337 cm^−1^ (symmetric *A_1_* ν(Fe−S*^b^*)), 353 and 364 cm^−1^ (components of asymmetric *T_2_* ν(Fe−S*^t^*)) and 398 cm^−1^ (symmetric *A_1_* ν(Fe−S*^t^*)). These bands are characteristic of protein-bound [4Fe-4S]^2+^ clusters and are readily assigned under idealized *T_d_* symmetry based on published isotope shifts and normal mode calculations [53].

### 2.5. Incorporation of an Fe-S cluster in At Apo-ISCA1a/2 via Cluster Transfer from At [2Fe-2S]^2+^-GRXS15

**Figure 9:**
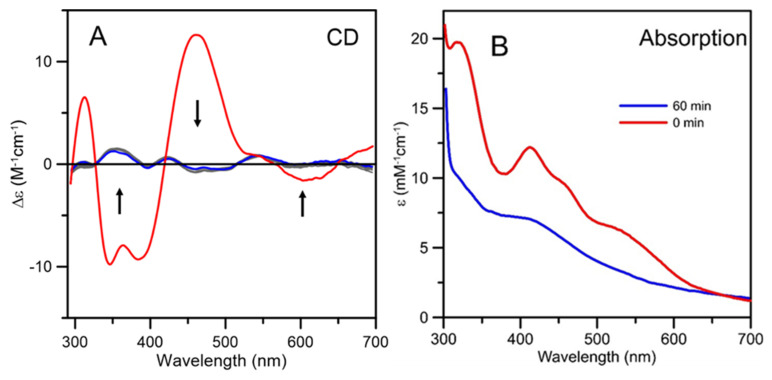
**Cluster transfer from *At* [2Fe-2S]^2+^-GRXS15 to *At* apo-ISCA1a/2 monitored by UV-visible absorption and CD spectroscopy as a function of time.** (**A**) The thick red line is the CD spectrum of [2Fe-2S]^2+^ cluster-bound GRXS15 before addition of At apo-ISCA1a/2 to the reaction mixture. The thin grey lines are CD spectra of the reaction mixture, GRXS15 (60 μM in [2Fe-2S]^2+^ clusters) mixed with DTT-pretreated apo-ISCA1a/2 (30 μM) in 100 mM Tris-HCl, pH 7.8, with 1 mM GSH, recorded at 1, 3, 5, 17, 20, 25, 28, 30, and 34 min after addition of apo ISCA1a/2. The thick blue line corresponds to the final CD spectra after 60 min. The arrows indicate the direction of change in CD intensity with time at selected wavelengths and Δε values are based on the initial concentration of [2Fe-2S]^2+^ clusters in the reaction mixture. (**B**) The thick red line is the absorption spectrum of [2Fe-2S]^2+^ cluster-bound GRXS15 before addition of *At* apo-ISCA1a/2 to the reaction mixture. The thick blue line corresponds to the final absorption spectrum 60 min after addition of *At* apo-ISCA1a/2 to the reaction mixture. ε values are based on the initial concentration of [2Fe-2S]^2+^ clusters in the reaction mixture.

Anaerobic cluster transfer from *At* [2Fe-2S]^2+^-GRXS15 to DTT-pretreated *At* apo-ISCA1a/2 in the presence of 1 mM GSH with a 2:1 donor:acceptor ratio was complete after < 1 min based on the loss of the intense CD spectrum of [2Fe-2S]^2+^-GRXS15, see Figure 9A. The resultant CD spectrum corresponded to [2Fe-2S]^2+^-ISCA1a/2, but the CD intensity indicated that only 20% of the original [2Fe-2S]^2+^ clusters are present as [2Fe-2S]^2+^-ISCA1a/2 (Figure 5 and Figure 9A). Since [4Fe-4S]^2+^-ISCA1a/2 has a negligible CD spectrum and the UV-visible absorption spectra of the [4Fe-4S]^2+^ and [2Fe-2S]^2+^ cluster-bound forms of ISCA1a/2 are quite distinct (see above and Figure 5) absorption can be used to monitor formation of [4Fe-4S]^2+^-ISCA1a/2 (Figure 9B). As for the CD spectra, no further change in the absorption spectra was observed after the first minute of reaction, and the absorption spectrum quantitatively showed that the remaining 80% of the original [2Fe-2S]^2+^ clusters were present as [4Fe-4S]^2+^-ISCA1a/2. Control experiments showed no change in the CD intensity of [2Fe-2S]^2+^-GRXS15 in the same reaction mixture without *At* ISCA1a/2 after 60 min. Overall, the results indicate that [2Fe-2S]^2+^ cluster transfer from [2Fe-2S]^2+^-GRXS15 to apo-ISCA1a/2 is a rapid reaction that results in a 80:20 mixture of [4Fe-4S]^2+^ and [2Fe-2S]^2+^ cluster-bound ISCA1a/2.

## 3. Discussion

**Figure 10:**
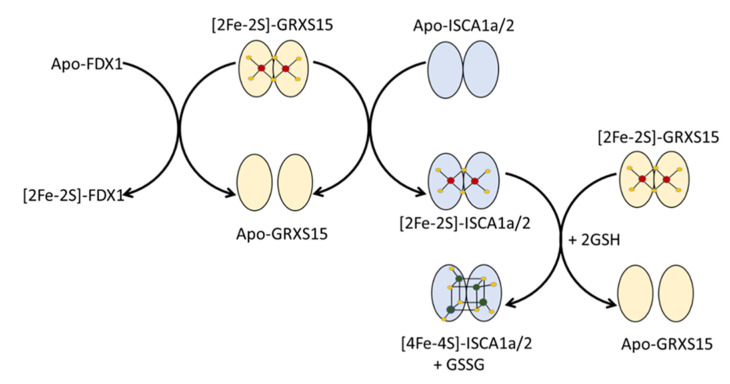
**Summary scheme for cluster trafficking between GRXS15 and its partner proteins**.

The maturation of all mitochondrial Fe-S proteins depends on the ISC machinery. The results presented above provide new insights into the function and properties of arabidopsis GRXS15 and ISCAs, two of the major classes of late-acting Fe-S cluster transfer/carrier proteins in plant mitochondria. In particular, the results show the interaction of GRXS15 with all three ISCA proteins, the ability of ISCA1 and 2 to form heterodimers, the type of Fe-S clusters incorporated by each protein and a mechanism for reductive [2Fe-2S]^2+^ to [4Fe-4S]^2+^ cluster conversion (Figure 10).

In vivo and in vitro studies have identified mitochondrial and bacterial monothiol GRXs as primary or sole acceptors of [2Fe-2S]^2+^ clusters assembled on U-type scaffold proteins [9,18,19]. However, important differences exist between plant and non-plant GRXs. Indeed, mitochondrial *At* GRXS15 is essential [27], unlike yeast Grx5. This suggests that GRXS15 has additional functions compared to its yeast ortholog. In zebrafish, the early lethality of a *grx5* mutant is due to IRP1 deregulation and a defect in heme synthesis [54]. However, there is no IRP1 ortholog in plants and heme synthesis occurs in plastids. In addition, poplar *GRXS15* failed to complement the yeast *∆grx5* mutant [43] and arabidopsis *GRXS15* only very partially complemented the yeast *∆grx5* mutant [27]. One explanation could lie in significant differences in the primary sequences of yeast and plant mitochondrial ATCs (Figure S2). For example, yeast Isa1/2 have large sequence insertions, which could hamper interaction with GRXS15. However, such complementation worked with GRX from various other sources [55]. Another explanation could be the existence of unusual properties or significant structural differences in plant mitochondrial GRXS15 compared to yeast and human orthologs. Based on the primary sequences, the major difference between GRXS15 and human and yeast Grx5 is the presence of an acidic N-terminal extension in GRXS15 (Figure S1). We have previously shown that the acidic N-terminal extension does not affect interaction with the mitochondrial BOLA4 [56] and have now determined that it does not prevent interaction with ISCA proteins. Another noteworthy primary sequence difference is the lack of a second semi-conserved cysteine in *At* GRXS15 (Figure S1). This cysteine forms a disulfide with the active site CGFS cysteine in yeast apo-Grx5 [57] and is required for [4Fe-4S]^2+^ cluster binding by yeast Grx5 in the absence of GSH [44]. This provides rationalization of the observed inability of *At* GRXS15 to reconstitute an Fe-S cluster in the absence of GSH, both in the presence of DTT or in samples pretreated with DTT.

Fe-S cluster reconstitution of *At* apo-GRXS15 in the presence of GSH resulted in samples containing a mixture of [2Fe-2S]^2+^ and linear [3Fe-4S]^1+^ clusters. As noted previously, such cluster mixtures are commonly found in as-purified and reconstituted samples of monothiol GRXs [44]. However, reconstituted *At* GRXS15 could be resolved into a pure [2Fe-2S]^2+^ cluster-containing fraction and a mixed [2Fe-2S]^2+^ and linear [3Fe-4S]^1+^ cluster-containing fraction using a Mono-Q column. This facilitated quantitative, CD-monitored cluster transfer studies between [2Fe-2S]-GRXS15 and apo-mFDX1, which revealed a complete, intact and unidirectional [2Fe-2S]^2+^ cluster transfer with a second order rate constant of 1.1 × 10^4^ M^−1^min^−1^ at room temperature. This cluster transfer has been reported in a previous study using native gels and CD spectroscopy, but the cluster content of *At* GRXS15 was not determined and only qualitative data were reported [27]. Overall, the results presented herein show that *At* GRXS15 is effective in [2Fe-2S] cluster trafficking and the maturation of [2Fe-2S] cluster-containing client proteins.

Mitochondrial ATC proteins in yeast (Isa1 and Isa2) and human (ISCA1 and ISCA2) function in the maturation of [4Fe-4S]^2+^ cluster-containing proteins [24,29,31,35]. In contrast, the role of mitochondrial ATC proteins in plants (ISCA1a, ISCA1b, ISCA2) has not been assessed and is solely based on yeast complementation studies, which showed that *At* ISCA1a or ISCA1b rescued the growth defects of Δ*Isa1* yeast cells and that *At* ISCA2 rescued the growth defects of Δ*Isa2* yeast cells, but not vice-versa [39]. In agreement with this apparent requirement of a heterodimer, we have only detected interaction between ISCA1a or ISCA1b and ISCA2 in Y2H, and not observed the formation of ISCA1 or ISCA2 homodimers.

In addition to the absence of functional studies, plant mitochondrial ATC proteins have never been purified and characterized prior to the work presented herein. Coexpressed *At* ISCA1a/2 purified as a stable heterodimer containing one [2Fe-2S]^2+^ cluster, which could be converted to a form containing one [4Fe-4S]^2+^ cluster per heterodimer by anaerobic Fe-S cluster reconstitution. The results presented herein demonstrate that [4Fe-4S]^2+^ cluster-bound *At* ISCA1a/2 heterodimer was also formed via rapid [2Fe-2S]^2+^ cluster transfer from *At* [2Fe-2S]-GRXS15 to *At* apo-ISCA1a/2 in the presence of excess GSH, but in the absence of DTT or IBA57. Since GRXS15 does not bind a [4Fe-4S]^2+^ cluster, [4Fe-4S]^2+^ cluster formation must involve the ISCA1a/2 heterodimer acting as a [4Fe-4S]^2+^ cluster assembler complex. The observation that the cluster transfer product is a 80:20 mixture of [4Fe-4S]-ISCA1a/2 and [2Fe-2S]-ISCA1a/2 heterodimers suggests that the first step involves intact [2Fe-2S]^2+^ cluster transfer from GRXS15 to form a [2Fe-2S]-ISCA1a/2 heterodimer. The second step is likely to involve binding of [2Fe-2S]-GRXS15 to [2Fe-2S]-ISCA1a/2 resulting in two [2Fe-2S]^2+^ clusters in close enough proximity for two-electron reductive coupling mediated by GSSG disulfide formation involving released or exogenous GSH (see Figure 10). Two-electron reductive coupling of two [2Fe-2S]^2+^ clusters to form a [4Fe-4S]^2+^ cluster has been well established and rationalized for bacterial ^Nif^IscA and IscU proteins using DTT and dithionite, respectively, as exogenous reducing agents [21,23]. Furthermore, cluster transfer from human [2Fe-2S]-GRX5 to the human apo-ISCA1/2 heterodimer also resulted in the formation of [4Fe-4S]-ISCA1/2 in the presence of excess DTT and GSH, as assessed by the combination of NMR, mass spectrometry (MS) and UV-visible absorption data [24]. This suggests that DTT likely had no deleterious effect in these Fe-S cluster transfer experiments and that it was not mandatory because of the presence of GSH. In another study, the role of three conserved cysteines of ISCA was clarified, showing that the C-terminal cysteines present in a CxC motif are required first to remove the [2Fe-2S]^2+^ cluster from the GRX donor [25]. The [2Fe-2S]^2+^ cluster rearrangement in the ISCA1/2 heterodimer, involving the third cysteine of each monomer and positioned approximately 65 residues upstream in the primary sequence, would liberate two cysteines of this C-terminal tail that become available to initiate extraction of the second [2Fe-2S]^2+^ cluster from GRX [25]. Hence, the results presented in this work add further support to the proposed role of ISCA1/2 heterodimers in mitochondrial Fe-S cluster biosynthesis as effectors of [2Fe-2S]^2+^ to [4Fe-4S]^2+^ cluster conversions using [2Fe-2S]^2+^ clusters supplied by a monothiol GRX and electrons necessary for the reductive coupling of these two [2Fe-2S]^2+^ clusters provided by glutathione. These results do not support a role for the GRXS15- and ISCA-interacting maturation factors, namely BOLA and IBA57, respectively, in [4Fe-4S]^2+^ cluster assembly on ISCA1a/2 heterodimers. Moreover, the O_2_ sensitivity of [4Fe-4S]-ISCA1a/2 indicates that it is unlikely to be functional under aerobic or oxidative stress conditions.

## 4. Materials and Methods

All the chemicals and materials were purchased from commercial suppliers (Fisher Scientific, Sigma-Aldrich Chemical Co, and GE Healthcare/Invitrogen) and were used without any further treatment unless otherwise stated.

### 4.1. Binary Yeast Two-Hybrid Assays

Yeast two-hybrid assays were carried out in the Gal4-based yeast two hybrid reporter strain CY306 [58]. The sequences encoding GRXS15 and ISCA proteins devoid of their mitochondrial targeting sequences were cloned into the pGADT7 or pGBKT7 vector (Clontech) between the NdeI or NcoI and BamHI restriction sites (primers used are listed in the Table S1). Gene products resulted in a protein fusion with the Gal4 activation domain (AD) or Gal4 DNA binding domain (BD), respectively. Transformants were selected on yeast nitrogen base (YNB) medium (0.7% yeast extract w/o amino acids, 2% glucose, 2% agar) without tryptophan and leucine (-Trp-Leu). Interactions were observed as cells growing on YNB medium in the absence of histidine (-His-Trp-Leu) at 30 °C. The strength of the interactions was evaluated by challenging growth in the presence of 2 or 5 mM of the competitive inhibitor of *HIS3* gene product 3-amino-1,2,4-triazole (3-AT). Images were taken five days after dotting (7µL per dot at an optical density of 0.05 at 600 nm). Results are representative of at least three independent experiments, each on two colonies per transformation event. All constructs producing fusion proteins were also assayed in control experiments after cotransformation with either a pGADT7 or pGBKT7 empty vector. 

### 4.2. Bimolecular Fluorescence Complementation

The full-length open reading frames coding for GRXS15 and ISCAs were amplified from *A. thaliana* leaf cDNAs with the primers presented in Table S1 and cloned in both pUC-SPYCE and pUC-SPYNE vectors using XbaI or BamHI and XhoI containing primers [59]. The constructs, placed under the control of a CaMV 35S promoter, consist of fusions of the proteins of interest at the N-terminus of nonfluorescent C- and N-terminal halves of YFP, respectively. Arabidopsis protoplasts were prepared and cotransfected with pUC-SPYNE and pUC-SPYCE construct pairs for 5 min in a PEG-based medium as described in [60] without vacuum infiltration. Pairs of constructs involving one empty vector were also transfected to control that none of the protein assayed could restore YFP fluorescence in the absence of interacting partners. Prior confocal analyses, fluorescent staining of the mitochondria within cells was performed by incubating freshly transfected protoplasts in a W5 solution [60] containing 100 nM MitoTracker^®^ Orange CMXRos (Invitrogen). The YFP fluorescence in arabidopsis protoplasts was recorded between 520 and 550 nm with a SP8 laser scanning confocal microscope (Leica Microsystems, Wetzler, Germany) after excitation with an argon laser at 514 nm. MitoTracker^®^ Orange CMXRos fluorescence was recorded between 580 and 620 nm after excitation at 560 nm. Leica LASX software was used to obtain images with and without maximum Z-stack intensity projection. Images were processed using the Adobe Photoshop software package. Results are representative of three independent bombardment experiments including the analysis of 10 to 20 cells per transformation event.

### 4.3. Analytical and Spectroscopic Methods

Protein concentrations were determined by the DC protein assay (Bio-Rad) using bovine serum albumin (Roche) as standard. Iron concentrations were determined colorimetrically using bathophenanthroline under reducing conditions after digesting proteins in 0.8% KMnO_4_/0.2 M HCl. A calibration curve was constructed from a series of dilutions of a 1000 ppm atomic absorption iron standard.

The preparation and handling of anaerobic samples for spectroscopic studies and cluster transfer experiments were carried out inside a Vacuum Atmospheres glove box under argon atmosphere at an oxygen level of 2 ppm or below. UV-visible absorption spectra were recorded in sealed quartz cuvettes at room temperature using a Shimadzu-3101PC spectrophotometer. CD spectra were recorded in sealed quartz cuvettes using a Jasco J-715 spectropolarimeter. Resonance Raman samples were prepared under strictly anaerobic conditions and comprised 18-μL frozen droplets of protein solutions (~2 mM in Fe-S clusters) mounted on the cold finger of an Air Products Displex Model CSA-202E closed cycle refrigerator (Air Products, Allentown, PA, USA). Resonance Raman spectra were recorded at 17 K using an Instrument SA Ramanor U1000 scanning spectrometer coupled with a Coherent Sabre argon ion laser. Spectra were recorded by photon counting for 1 s every 0.5 cm^−1^, using 7 cm^−1^ resolution, and each spectrum was the sum of 80–120 scans. The spectroscopic data presented in this work were representative of a single experiment repeated at least three times with the same results. Kinetic analyses and simulations were carried out using the IBM Kinetiscope kinetics simulation software. 

### 4.4. Overexpression, Aerobic Purification and Fe-S Cluster Reconstitution of At GRXS15

The sequence coding the mature form of *At* GRXS15 was amplified from *A. thaliana* leaf cDNA using primers listed in Table S1 and cloned into the NcoI-BamHI restriction sites of pET3d. *At* GRXS15 was heterologously expressed in an *E. coli* BL21 (DE3) containing the pSBET plasmid, which allows expression of the tRNA needed to recognize the AGG and AGA rare codons. One colony was grown overnight at 37 °C in 100 mL LB medium containing 100 μg/mL ampicillin and 30 μg/mL kanamycin. 1 L of the same medium was inoculated by adding 20 mL of the culture grown overnight and then incubated at 37 °C until exponential growth phase. Protein expression was induced by adding isopropyl 1-thio-β-D-galactopyranoside (IPTG) to a final concentration of 100 μg/mL. The cells were allowed to grow for an additional 5 h at 34 °C before harvesting by centrifugation at 6690× *g* and storing at −80 °C for later use.

For aerobic purification of *At* GRXS15, 20 g of cell paste were resuspended in 50 mL of buffer A (100 mM Tris-HCl, pH 7.8) containing 2 mM GSH, 150 µg/mL PMSF, 2 mU/mL DNase (Roche), and 0.5 µg/mL RNase (Roche), and lysed by intermittent sonication on ice. After breaking the cells, the soluble and the insoluble fractions were separated by centrifugation at 39800× *g* at 4 °C for 1 h. The soluble fraction was subjected to a 40% ammonium sulfate cut, and the precipitate was removed by centrifugation at 39800× *g* for 30 min. The supernatant containing *At* GRXS15 was loaded onto a phenyl sepharose column equilibrated with buffer A containing 2 mM GSH and 1.0 M ammonium sulfate and eluted by a decreasing linear gradient of 1.0 to 0 M ammonium sulfate. Based on gel electrophoresis, fractions containing GRXS15 were concentrated using Amicon ultrafiltration with a YM10 membrane. The concentrated fraction was loaded onto a 25 mL Q Sepharose anion exchange column, previously equilibrated with buffer A, and eluted with an increasing linear gradient of 0 to 1.0 M sodium chloride. Apo-GRXS15 was prepared by incubating as-isolated samples with a 50-fold excess of EDTA and a 20-fold excess of potassium ferricyanide for 60 min. Apo-GRXS15 was then purified with a 15 mL desalting column to remove residual iron and sulfide under anaerobic conditions. 

Reconstitution of Fe-S clusters on apo-GRXS15 was carried out under anaerobic conditions inside a glove box. In the presence of 5 mM GSH, apo-GRXS15 was incubated with 12-fold excess of FAS, 12-fold excess of L-cysteine and catalytic amounts of the *E. coli* cysteine desulfurase IscS, for approximately 2 h in a strictly anaerobic environment. The cluster-bound GRXS15 was loaded on to a 5 mL Mono-Q column to remove excess reagents and eluted with an increasing salt gradient of 0–1.0 M NaCl. The Mono-Q column was able to separate Fe-S cluster-bound GRXS15 into two different colored fractions.

### 4.5. Overexpression and Anaerobic Purification of His-Tagged At mFDX1 

The cloning of *At* mFDX1 (At4g05450) in pET15b was described previously [27]. Recombinant mFDX1 was expressed in an *E. coli* BL21 (DE3) strain. A single colony was grown in LB medium at 37 °C and exponential protein overexpression was induced with IPTG to a final concentration of 100 μg/mL. The bacterial culture was further allowed to cultivate at 37 °C for 5–6 h. The dark reddish-brown cells were harvested by centrifugation at 6690× *g* at 4 °C and stored at −80 °C for later use. 

For the aerobic purification of mFDX1, 20 g of cell paste were thawed and resuspended in 50 mL of buffer A and lysed by intermittent aerobic sonication on ice. After cell lysis, the soluble and the insoluble fractions were separated by centrifugation at 39800× *g* at 4 °C for 2 h. The dark reddish-brown soluble fraction containing *At* mFDX1 was then loaded onto a 25 mL His-Trap HP column previously equilibrated with binding buffer (100 mM Tris-HCl, pH 7.8, containing 0.5 M NaCl and 20 mM imidazole). The column was washed with 10 column volumes of binding buffer before the protein of interest was eluted with a 20–500 mM imidazole gradient. The purest fractions containing *At* holo-mFDX1 were collected, and imidazole was removed by loading mFDX1 onto a 25 mL desalting column. *At* apo-mFDX1 was prepared by acid precipitation with 10% trichloroacetic acid. The pelleted mFDX1 was resuspended in buffer A and dialyzed 4–5 times by ultrafiltration dialysis using a YM10 membrane with the same buffer. The *At* apo-mFDX1 used for spectroscopic studies was >95% pure, based on SDS-PAGE gels.

### 4.6. Overexpression, Anaerobic Purification, and Fe-S Cluster Reconstitution of His-Tagged At ISCA1a/2 and ISCA1b/2

The sequences coding the mature forms of ISCA1a or ISCA1b were cloned in pET28a and the one of ISCA2 in pET12a between the NdeI-BamHI restriction sites. For cloning in pCDF Duet, ISCA1a was subcloned from pET28a using the NcoI-BamHI restriction sites of pCDF Duet in which ISCA2 was cloned in the NdeI-XhoI restriction sites. All primers are listed in Table S1.

*E. coli* BL21 (DE3) cells harboring the pCDFDuet ISCA1a/2 plasmid were cultivated overnight at 37 °C in LB medium containing 100 μg/mL spectinomycin, and 20 mL of the culture grown overnight were used to inoculate 1 L of the same medium. Protein expression was induced with IPTG to a final concentration of 100 μg/mL when OD_600_ was between 0.6–0.8. The cells were allowed to grow for an additional 5 h at 34 °C before harvesting by centrifugation at 6690× *g* and storing at −80 °C for later use. 

The procedure used to purify ISCA1a/2 was very similar to the purification procedure of mFDX1, except that sonication and all chromatographic processes were carried out in the glove box under anaerobic conditions (O_2_ < 2 ppm). The buffers used in the purification procedure were rigorously degassed to remove oxygen. For these purifications, 18 g of reddish cell pellets were thawed and resuspended in 30 mL of buffer A containing 150 µg/mL PMSF, 2 mU/mL DNase (Roche) and 0.5 µg/mL RNase (Roche), and lysed by intermittent anaerobic sonication on ice. After breaking the cells, the soluble and the insoluble fractions were separated by centrifugation at 39800× *g* at 4 °C for 1.5 h. The soluble fractions were loaded onto a 25 mL His-Trap HP column, which was pre-equilibrated with binding buffer. The column was washed with 10 column volumes of binding buffer before the protein of interest was eluted with a 20–500 mM imidazole gradient. The purest fraction containing [2Fe-2S]^2+^ cluster-bound heterodimeric *At* ISCA1a/2 was collected, and imidazole was removed by loading the concentrated ISCA1a/2 fraction onto a 25 mL desalting column. Apo-ISCA1a/2 was prepared by treating the holo-protein with a 50-fold excess of EDTA and a 20-fold excess of potassium ferricyanide under anaerobic conditions and removing the excess reagents by ultrafiltration dialysis using a YM10 membrane to remove excess iron and sulfide.

Reconstitution of Fe-S clusters on as-purified ISCA1a/2 was carried out under anaerobic conditions in the presence of 2 mM DTT, a 12-fold excess of FAS, 12-fold excess of L-cysteine, and catalytic amounts of IscS, for approximately 5 h in a strictly anaerobic environment. The reconstitution mixture was loaded on to a 10 mL Hitrap Q-Sepharose column (GE Healthcare) and proteins were eluted with an increasing salt gradient of 0–1.0 M NaCl. A single colored fraction containing predominantly [4Fe-4S]^2+^ cluster-bound ISCA1a/2 was eluted under an increasing NaCl gradient.

### 4.7. Protocol for Donor-to-Acceptor Cluster Transfer Studies Monitored by UV-Visible CD Spectroscopy

The time courses of Fe-S cluster transfer from the cluster-bound donor to apo-acceptor were monitored at room temperature under anaerobic conditions in 1-cm cuvettes using CD spectroscopy. In all cases, the apo-acceptor protein was incubated with 2 mM DTT for 30 min and buffer washed anaerobically three times using centricon ultrafiltration to remove DTT (DTT pretreatment), prior to initiation of the reaction by the addition of apo-protein to the donor protein solution. The CD spectrum was monitored until no further change was observed. Peak-to-trough or fixed wavelength changes in CD intensity were used to assess the extent of cluster transfer as a function of time. The data were fitted to second order kinetics using the Kinetiscope chemical kinetics simulator software package (IBM), based on the initial concentration of Fe-S clusters on the donor protein and the concentration of the apo-acceptor protein. The directionality of cluster transfer was assessed by repeating the reaction with cluster-bound acceptor as the donor and the apo-donor as the acceptor. The lability of the Fe-S center in the donor in the reaction mixture was assessed by monitoring the UV-visible absorption or CD spectrum of the donor, in the absence of the acceptor, over the time course of the reaction. The specific conditions for each cluster transfer reported in this work are given in the results section and in the figure legends.

## Figures and Tables

**Table 1:** Fe-S stretching frequencies (cm^−1^) and vibrational assignments for the [2Fe-2S]^2+^ clusters in *A. thaliana* ISCA1a/2, *A. vinelandii* ^Nif^IscA and *Spinacia oleracea* ferredoxin.

Assignments Under D_2h_ Symmetry ^a^	*S. oleracea* 2Fe Ferredoxin ^b^	*A. vinelandii* ^Nif^IscA ^b^	*A. thaliana* ISCA1a/2
B_2u_^b^	427	421	421
A_g_^b^	395	396	398
B_3u_^b^	367	358	368
B_1u_^t^, B_2g_^t^	357	345	352
A_g_^t^	338	338	334
B_1g_^b^	329	325	324
B_3u_^t^	283	290	288

^a^ Symmetry labels assuming idealized D_2h_ symmetry for the Fe_2_S_2_^b^S_4_^t^ core, where Fe-S^b^ and Fe-S^t^ indicate bridging and terminal stretching, respectively. ^b^ Taken from Ref [23].

## Supplementary Materials

Supplementary materials can be found at https://www.mdpi.com/1422-0067/21/23/9237/s1.

## Abbreviations

At	Arabidopsis thaliana
Sc	Saccharomyces cerevisiae
Hs	Homo sapiens
ISC	iron-sulfur cluster assembly
Fe-S	iron-sulfur
S^b^	bridging sulfide
S^t^	terminal cysteinyl sulfur
ATC	A-type carrier
CD	circular dichroism
RR	resonance Raman
DTT	dithiothreitol
DT	dithionite
GSH	glutathione
IPTG	isopropyl 1-thio-β-D-galactopyranoside
FAS	ferrous ammonium sulfate
PMSF	phenylmethanesulfonyl fluoride
SDS-PAGE	sodium dodecyl sulfate polyacrylamide gel electrophoresis
TCA	trichloroacetic acid
