# Supplementary material for: The Arabidopsis Mitochondrial Glutaredoxin GRXS15 Provides [2Fe-2S] Clusters for ISCA-Mediated [4Fe-4S] Cluster Maturation
Source: Int J Mol Sci. 2020 Dec 3;21(23):9237. doi: 10.3390/ijms21239237 (PMC7730481; doi:10.3390/ijms21239237)
Supplement: Supplementary file 1 [file ijms-21-09237-s001.pdf]

Supporting information for the manuscript

The Arabidopsis mitochondrial glutaredoxin GRXS15 provides [2Fe-2S] clusters for ISCA-mediated [4Fe-4S] cluster maturation

Tamanna Azam[1,†], Jonathan Przybyla-Toscano[2,†], Florence Vignols[3], Jérémy Couturier[2], Nicolas Rouhier[2], and Michael K. Johnson[1,*]

Figure S1. Amino acid sequence alignment of the mature region of mitochondrial monothiol glutaredoxins from *Arabidopsis thaliana*, *At* GRXS15 ; *Saccharomyces cerevisiae*, *Sc* Grx5 ; and human, *Hs* GLRX5.

Figure S2. Amino acid sequence alignment of ATCs.

Figure S3. BiFC assays between Arabidopsis GRXS15 and ISCA proteins in Arabidopsis leaf protoplasts (complement).

Figure S4. Control studies supporting unidirectional [2Fe-2S] cluster transfer from GRXS15 to mFDX1.

Figure S5. Oligomeric state of as-purified *At* ISCA1a/2 heterocomplex assessed by size exclusion chromatography and gel electrophoresis.

Table S1. Primers used in this study.

```
AtGRXS15        SSTVPSDSDTHDDFKPTQKVPPDSTDSLKDIVENDVKDNPVMIYMKGVPESPQCGFSSLA
HsGRX5          ---------------------GAGGGGSAEQLDALVKKDKVVVFLKGTPEQPQCGFSNAV
ScGRX5          -----------------------LSTEIRKAIEDAIESAPVVLFMKGTPEFPKCGFSRAT
                                                        *    ** ** * ****

AtGRXS15        VRVLQQYNV-P--ISSRNILEDQELKNAVKSFSHWPTFPQIFIKGEFIGGSDIILNMHKE
HsGRX5          VQILRLHGVRD--YAAYNVLDDPELRQGIKDYSNWPTIPQVYLNGEFVGGCDILLQMHQN
ScGRX5          IGLLGNQGVDPAKFAAYNVLEDPELREGIKEFSEWPTIPQLYVNKEFIGGCDVITSMARS
                   *    *        * * * **    *  * *** **     ** **↑*    *

AtGRXS15        GELEQKLKD-------VSGNQD
HsGRX5          GDLVEELKKLGIHSALLDEKKDQDSK
ScGRX5          GELADLLEE--AQALVPEEEEETKDR
                * *   *
```

Figure S1. **Amino acid sequence alignment of the mature region of mitochondrial monothiol glutaredoxins from *Arabidopsis thaliana*, *At* GRXS15 ; *Saccharomyces cerevisiae*, *Sc* Grx5 ; and human, *Hs* GLRX5.** The sequence alignment was performed with MUSCLE. Conserved amino acids are indicated with a star and the semi-conserved cysteine residue present in the C-terminal region of some monothiol GRXs by an arrow. The unusual N-terminal extension found in plant GRXS15 sequences is boxed,

```
ScIsa2                           MQAKLLFTRLNFRRPSTTLRQFPLTCFLFHSK--AFYSDLVTK
AtISCA2                                    MSRSLVKRVAPYLAGRIRENHRLLNFSSASAIK
HsISCA2                            MAAAWGSSLTAATQRAVTPWPRGRLL---------TASLGP
HsISCA1                   MVAAGGGARTEGAVRRSLWRQCARRVHGEKLRRPTFGPRHRGAGTAKMSA
ScIsa1          MINTGRSRNSVLLAHRFLSTGGFWRGGTNGTMSRTINNVNPFKLKFIPKTVPAAADSVSP
AtISCA1a                                                    MKASQILAAAAARVGP
AtISCA1b

ScIsa2          EPLITPKRIINK------------------------------------------------
AtISCA2         EASSSSSSQPES------------------------------------------------
HsISCA2         QARREASSSSPE------------------------------------------------
HsISCA1         SLVRATVRAVSK------------------------------------------------
ScIsa1          DSQRPGKKPFKFIVSNQSKSSKASKSPKWSSYAFPSRETIKSHEEAIKKQNKAIDEQIAA
AtISCA1a        A-----------------------------------------------------------
AtISCA1b

ScIsa2          ---------------------TPGLNLSISERASNRLAEIYRNSKEN--LRISVESGGCH
AtISCA2         ---------------------SSNDVVHLSDNCIRRMKELQSSEPEKKMLRLGVETGGCS
HsISCA2         ---------------------AGEGQIRLTDSCVQRLLEITEGSE---FLRLQVEGGGCS
HsISCA1         -----------------RKLQPTRAALTLTPSAVNKIKQLLKDKPEHVGVKVGVRTRGCN
ScIsa1          AVSKNDCSCTEPPKKRKRKLRPRKALITLSPKAIKHLRALLA-QPEPKLIRVSARNRGCS
AtISCA1a        ---------------------LRKQVLTLTDEAASRVHHLLQQRQKP-FLRLGVKARGCN
AtISCA1b                             MRKQVLALSDTAAARIRQLLQHQQKP-FLRLAVEAKGCN
                                                                         **

ScIsa2          GFQYNLTLEPATKPDIKNDVKDKEFSDDLDDDDSKDIIYVLPEDKGRVIIDSKSLNILNN
AtISCA2         GFQYKF-----------------ELDNRTNPDDR-----VFEKNGVKLVVDNVSYDLVKG
HsISCA2         GFQYKF-----------------SLDTVINPDDR-----VFEQGGARVVVDSDSLAFVKG
HsISCA1         GLSYTL-----------------EYTKTKGDSDE-----EVIQDGVRVFIEKKAQLTLLG
ScIsa1          GLTYDL-----------------QYITEPGKFDE-----VVEQDGVKIVIDSKALFSIIG
AtISCA1a        GLSYTL-----------------NYADEKGKFDE-----LVEEKGVRILVEPKALMHVIG
AtISCA1b        GLSYVL-----------------NYAQEKGKFDE-----VVEEKGVKILVDPKAVMHVIG
                *  *                            *

ScIsa2          TTLTYTNELIGSSF-KIINGSLKSSCGCGSSFDIEN
AtISCA2         ATIDYEEELIRAAFVVAVNPSAVGGCSCKSSFMVKL
HsISCA2         AQVDFSQELIRSSFQVLNNPQAQQGCSCGSSFSIKL
HsISCA1         TEMDYVEDKLSSEF-VFNNPNIKGTCGCGESFNI
ScIsa1          SEMDWIDDKLASKF-VFKNPNSKGTCGCGESFMV
AtISCA1a        TKMDFVDDKLRSEF-VFINPNSQGQCGCGESFMTTSTSSAKQSAS
AtISCA1b        TEMDFVDDKLRSEF-VFVNPNATK-CGCGESFTTT
                             *    *      * *  **
```

Figure S2. Amino acid sequence alignment of ATCs. Amino acid sequence alignment of the A-type carrier (ATC) proteins from *Arabidopsis thaliana* (*At*); *Saccharomyces cerevisiae* (*Sc)*; and human (*Hs).* The sequence alignment was performed with MUSCLE and highlights the insertions present in yeast Isa1 and Isa2. Conserved amino acids are indicated with an asterisk.

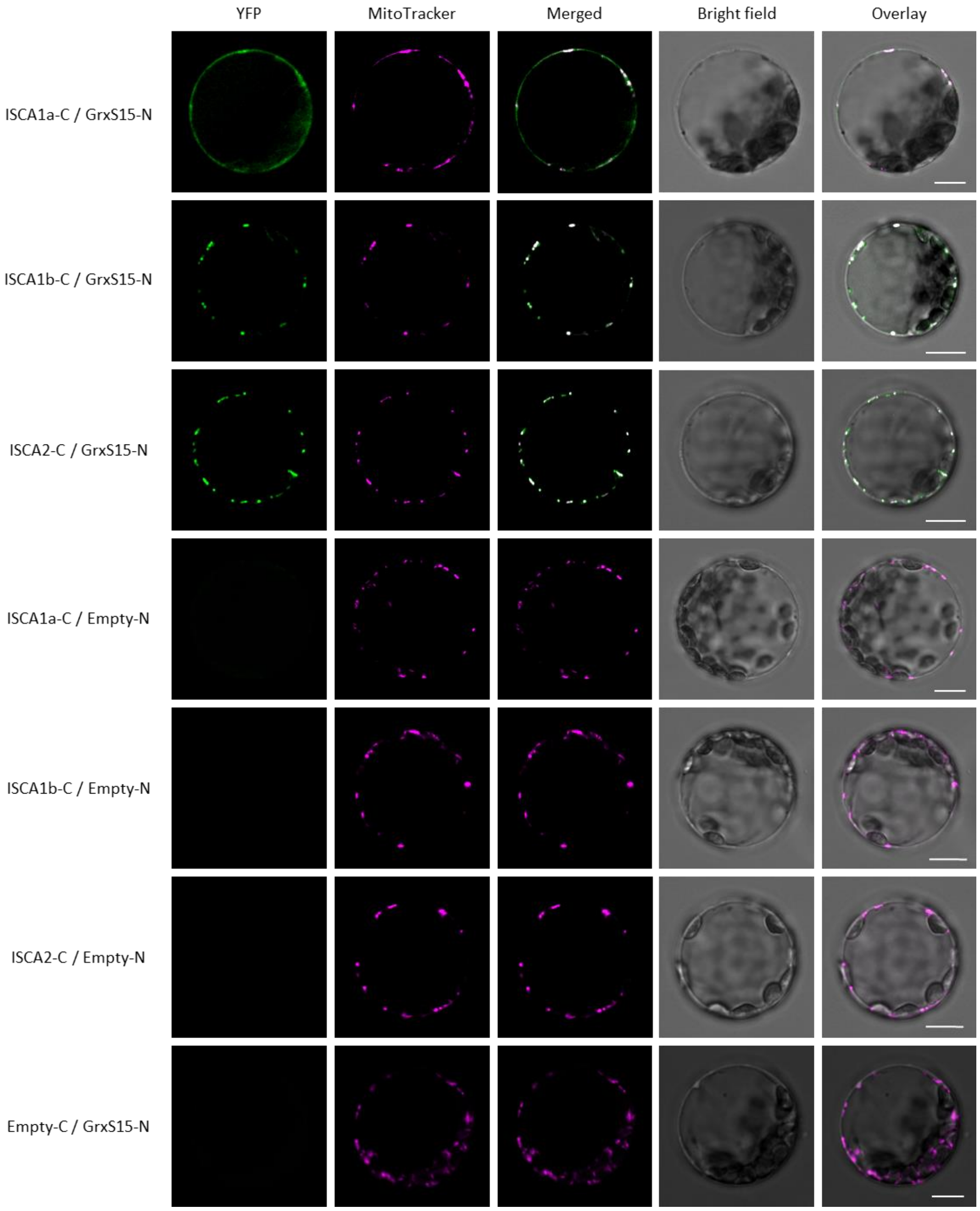

Figure S3. BiFC assays between Arabidopsis GRXS15 and ISCA proteins in Arabidopsis leaf protoplasts (complement). *Arabidopsis* protoplasts obtained from 4-week-old plantlets were transfected with combinations of two vectors expressing GRXS15 fused to the N-terminal region of the YFP protein (GRXS15-N in panels) and ISCAs cloned upstream of the C-terminal region of YFP (ISCA-C in panels). The YFP fluorescence was recorded 24h post-transfection by confocal microscopy. All confocal images shown here were captured at selected confocal plans without Z-stack intensity projection. Negative controls verifying that none of the proteins expressed alone can restore YFP fluorescence are shown. BiFC results obtained using opposite protein fusion conformations (GRXS15-C co-expressed with ISCA-N) showed less clear-cut results as a strong fluorescence in the cytosol suggested the formation of aggregates was obtained for some combinations. YFP, yellow fluorescent protein. Bars = 10 µm.

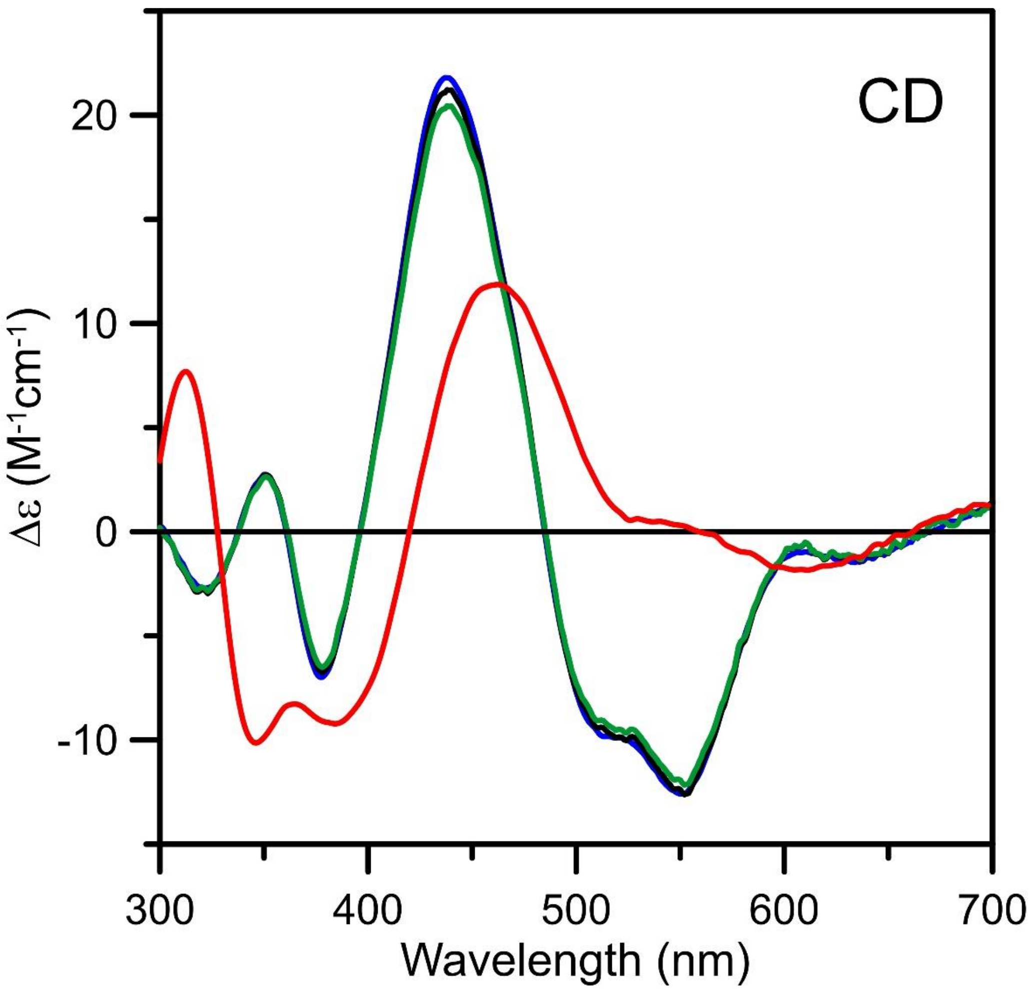

Figure S4. Control studies supporting unidirectional [2Fe-2S] cluster transfer from GRXS15 to mFDX1. Attempted [2Fe-2S] cluster transfer from *At* holo-mFDX1 to *At* apo-GRXS15 monitored by CD spectroscopy as a function of time. CD spectra of the reaction mixture that was initially 40 µM in holo-mFDX1 and 80 µM in apo-GRXS15 monomers. The blue line corresponds to holo-mFDX1 recorded before addition of apo-GRXS15. The black and green lines correspond to the CD spectra recorded at 10 and 40 min, respectively, after the addition of apo-GRXS15. The red line corresponds to the CD spectrum expected for holo-GRXS15. The lack of a significant change in the CD spectrum after 40 min indicates no significant cluster transfer. The reaction conditions were the same as those used for the successful reverse cluster transfer shown in Fig. 4B and 4C.

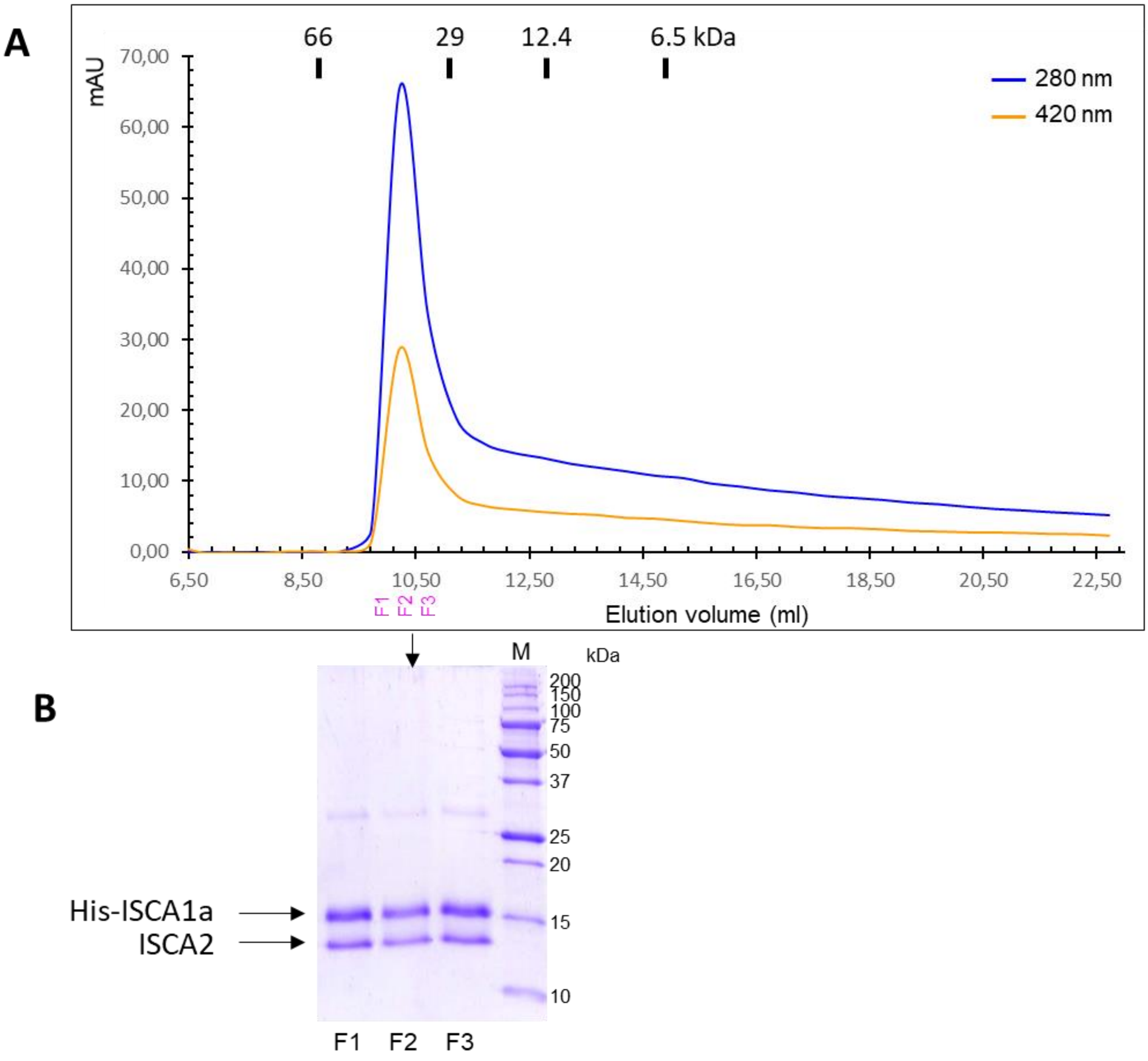

Figure S5. Oligomeric state of as-purified *At* ISCA1a/2 heterocomplex assessed by size exclusion chromatography and gel electrophoresis.

A. Representative elution profile of IMAC-purified ISCA1a/2 sample (100 µg) on analytical size-exclusion chromatography (Superdex™ 75 10/300 column) equilibrated with a buffer containing 30 mM Tris-HCl buffer pH 8.0 and 100 mM NaCl and connected to an ÄKTA-Purifier™ (GE Healthcare) at a temperature of 10°C. Absorbances at 280 and 420 nm were recorded. The apparent molecular mass has been estimated from the calibration curve established with molecular mass standards ranging from 6.5 to 66 kDa (Sigma). The molecular mass of each protein and their elution volume are as follows: aprotinin (bovine lung) - 6,500 Da - 14.99 ml; cytochrome C (horse heart) - 12,400 Da - 12.86 ml; carbonic anhydrase (bovine erythrocytes) - 29,000 Da - 11.12 ml, albumin (bovine serum) - 66,000 Da - 9.06 ml. B. Gel electrophoresis of the fractions corresponding to the sole peak of the elution profile. Proteins were separated by 15% SDS-PAGE and stained with Coomassie Brilliant blue. Molecular mass standards are indicated. Considering the respective theoretical molecular masses of 15.7 and 13.2 kDa for *At* ISCA1a and *At* ISCA2, we deduced that the 34 kDa apparent volume indicated that *At* ISCA1a/2 is a heterodimer.

AGI number	Gene names	Plasmids	Primer names	Sequences	N-terminal sequences
At3g15660	GRXS15	pET3d, pGADT7, pGBKT7	AtGRXS15 for	5’ CCCCCCATGGCTAGATTTTCCTCAACAGTGCCA 3’	MARFSSTVP
			AtGRXS15 rev	5’ CCCCGGATCCTCAATCTTGGTTTCCGGA 3’	
		pUC-SPYNE/SPYCE	AtGRXS15 for2	5’ CCCCGGATCCATGGCGGCTTCTTTATCG 3’	MAASLS
			AtGRXS15 rev2	5’ CCCCCTCGAGATCTTGGTTTCCGGAGAC 3’	
At2g16710	ISCA1a	pET28a, pCDFDUET pGADT7, pGBKT7	AtISCA1a for	5’ CCCCCCCCATATGAAGCAAGTATTAAC 3’	MKQVLT
			AtISCA1a rev	5’ CCCCGGATCCTTAACTAGCACTCTGCTT 3’	
		pUC-SPYNE/SPYCE	AtISCA1a for2	5’ CCCCTCTAGAATGAAAGCTTCTCAAATT3’	MKASQI
			AtISCA1a rev2	5’ CCCCCTCGAGACTAGCACTCTGCTTAGC3’	
At2g36260	ISCA1b	pET28a, pGADT7, pGBKT7	AtISCA1b for	5’ CCCCCCCCATATGCGAAAGCAAGTATTAGCA 3’	MRKQVLA
			AtISCA1b rev	5’ CCCCGGATCCTCATGTTGTCGTGAATGACTC 3’	
		pUC-SPYNE/SPYCE	AtISCA1bfor2	5’ CCCCTCTAGAATGAGAAAGCAAGTATTA 3’	MRKQVL
			AtISCA1b rev2	5’ CCCCCTCGAGTGTTGTCGTGAATGACTC 3’	
		mutagenesis	AtISCA1bV66V for	5’ GGTGTAAAGATCCTTGT**C**GATCCAAAGGCGGTG 3’	
			AtISCA1bV66V rev	5’ CACCGCCTTTGGATC**G**ACAAGGATCTTTACACC 3’	
At5g03905	ISCA2	pET12a, pGADT7, pGBKT7	AtISCA2 for	5’ CCCCCCCCATATGTCCCAACCTGAATCG 3’	MSQPES
			AtISCA2 rev	5’ CCCCGGATCCTCAGAGTTTCACCATGAA 3’	
		pCDFDUET	AtISCA2 rev2	5’ CCCCCTCGAGGAGTTTCACCATGAAGGA 3’	
		pUC-SPYNE/SPYCE	AtISCA2 for2	5’ CCCCTCTAGAATGTCAAGATCTCTGGTG 3’	MSRSLV
			AtISCA2 rev3	5’ CCCCCTCGAGGAGTTTCACCATGAAGGA 3’	

Table S1. Primers used in this study. Restriction sites required for cloning are in red. For nuclear-encoded plastidial proteins, the sequences coding for the putative targeting sequences have been removed for the cloning in pET and pGADT7/pGBKT7 vectors. Consequently, a methionine codon has been introduced in corresponding forward (for) primers. Owing to the use of NcoI restriction site for AtGRXS15 cloning, an alanine has been added after the methionine to respect the open reading frames. For ISCA1b, an internal BamHI restriction site was mutated by site-directed mutagenesis introducing a silent substitution in the AtISCA1bV66V forward and reverse primers (in bold). Full-length sequences have been cloned in pUC-SPYCE/SPYNE vector.
